# Quantifying the net effect of biodiversity on ecological stability

**DOI:** 10.1038/s41467-026-75047-z

**Published:** 2026-07-07

**Authors:** Charlotte Kunze, Maren Striebel, Dominik Bahlburg, Toni Schott, Ian Donohue, Helmut Hillebrand

**Affiliations:** 1 Institute for Chemistry and Biology of the Marine Environment (ICBM), School of Mathematics and Science, Carl von Ossietzky Universität Oldenburg, Oldenburg, Germany; 2 Helmholtz-Institute for Functional Marine Biodiversity at the University of Oldenburg (HIFMB), Oldenburg, Germany; 3 Alfred Wegener Institute, Helmholtz-Centre for Polar and Marine Research (AWI), Bremerhaven, Germany; 4 Department of Zoology, School of Natural Sciences, Trinity College Dublin, Dublin, Ireland

**Keywords:** Biodiversity, Community ecology, Climate-change ecology

## Abstract

Understanding the relationship between biodiversity and both the functioning and stability of ecosystems has been a central focus of ecologists for decades. A step-change in our understanding of the biodiversity–ecosystem functioning relationship was enabled by explicit measurement of the additional functioning provided by biodiversity through comparing expected and observed yields in multi-species communities. However, we lack an equivalent measure for stability. Here, we quantify the net biodiversity effect on stability using model simulations and a microcosm experiment that exposes different phytoplankton species and their combinations to temperature increases and fluctuations. As an emergent property of communities, stability frequently exceeds the expected stability of the combined component species, leading to a net biodiversity effect on stability analogous to the effect on functioning. In our simulations, these effects depend on the strength of competitive interactions as well as species composition and their thermal optima. Experimentally, the stabilising effect of biodiversity is, however, non-linear, greatest for two-species combinations, and varies with both community composition and disturbance regime. Quantifying the net biodiversity effect on ecological stability advances our mechanistic understanding of the biodiversity–stability relationship, and provides crucial information to support ecosystem management and conservation.

## Abstract (web summary)

Biodiversity shapes how ecosystems respond to environmental change. The current phytoplankton experiments and simulations show that biodiversity enhances ecosystem stability beyond expectations from monocultures, depending on species interactions and thermal trait alignment.

## Introduction

Exploring the intricate interplay between biodiversity and ecosystem stability has been a focal point of ecological research for decades, with a multitude of studies exploring the factors that underlie the stability of ecosystems^1^. Ecological stability comprises a family of measures that together encapsulate the dynamics of the system and its response to disturbance, and include the ability of a system to withstand a disturbance (resistance), recover from it (recovery), and capture how it varies over time (temporal stability)^2,3^.

Recent advances in modelling and synthesis have enhanced our understanding of how stability might be influenced by the diversity of species in a system^4–9^, demonstrating that there is no universal diversity–stability relationship. Rather, individual dimensions of stability differ in their relationships with biodiversity, depending on the nature of environmental change^8,10,11^. For example, increasing species richness decreases resistance to experimental drought in plant communities^10^. Similarly, increasing species richness in microbial communities decreases resistance to experimental warming but increases temporal stability^8^. This richness-dependent effect on resistance indicates that species-rich systems may be more likely to lose growth potential under disturbance if species growth is negatively correlated with disturbance tolerance^10,12^. By contrast, diversity has been found to stabilise community dynamics over time (that is, reduce temporal variability) when ecosystems are exposed to environmental variability^6,13^, even though individual populations within diverse communities might exhibit enhanced fluctuations^14^. Thus, a diverse community can buffer against environmental fluctuations because different species respond asynchronously to disturbances^15,16^.

Moreover, interspecific interactions can help stabilise or destabilise community dynamics^17^. Competition may promote compensatory dynamics—where declines in one species are offset by increases in another^18^—leading to strong stabilising effects of biodiversity^14,19^. By contrast, if competing species respond similarly to environmental change, strong competition can also synchronize population fluctuations or intensify declines after disturbance, producing destabilising effects^19^. Understanding to what extent stability emerges from biodiversity requires knowledge about how population stability relates to community stability in the context of disturbances. Distinguishing the role of individual species responses from the aggregated effects of interspecific interactions is therefore crucial to predict biodiversity–stability relationships in the context of environmental change^20^.

The related question of how biodiversity affects ecosystem functioning has benefited immensely from such a decomposition, which has become a fundamental element of understanding net biodiversity effects^21,22^. By quantifying expected functions based on species-specific performance in monoculture and comparing these expectations with observed rates in multi-species communities, Loreau and Hector^22^ highlight the dual importance of both species diversity (via complementarity) and species identity (via selection effects) in ecosystem functioning^22^. A recent framework that partitions biodiversity effects on the temporal stability of ecosystem functioning has also advanced our understanding by distinguishing interaction-dependent from interaction-independent effects of biodiversity on temporal stability^19^. What is currently lacking is an equivalent metric to quantify the additional stability provided by biodiversity in the context of environmental change that incorporates the multidimensional nature of ecological stability.

**Fig. 1:**
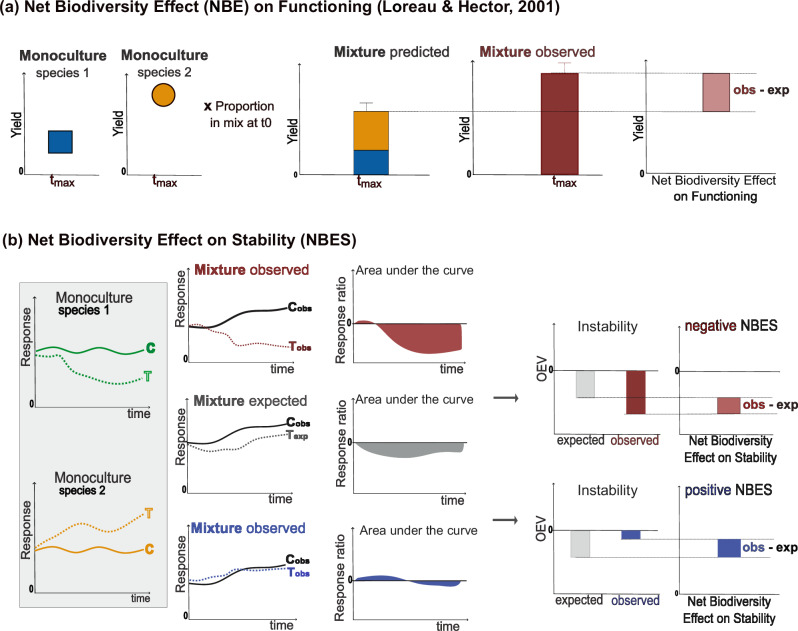
Conceptual demonstration of the quantification of the net biodiversity effect on functioning and stability as mean ± SE. The net biodiversity effect (NBE) on functioning is calculated as the difference between an observed and an expected biomass yield based on monospecific yields at the end of an experiment (**a**). The NBE on stability is calculated as the difference between observed and expected stability. Expected stability is determined from the sum of species-specific response ratios in monocultures and their observed contribution to biomass in the equivalent undisturbed control mixture. Observed stability is estimated from the realised biomass in treatment, abbreviated as *T*, relative to the undisturbed control, abbreviated as *C*. As an example, we have marked the area under the curve of the observed and expected response ratio [based on overall ecological vulnerability (OEV) in this example], which represents an integrative measure of (in)stability. Here, we concentrate on a typical, growth-reducing disturbance and decipher the two cases of lower observed stability than expected and thus a negative NBES (red colour), and higher observed stability than expected, and thus a positive NBES (blue colour) (**b**). Please note that this figure is purely conceptual and does not use any data.

Drawing from Loreau and Hector’s^22^ approach for quantifying the net biodiversity effect on ecosystem functioning, we here introduce an approach to assess the added stability provided by biodiversity in the face of disturbance and considering the multidimensional nature of stability. Specifically, our approach enables quantification of the net biodiversity effect on functional stability (NBES)—that is, the stability of an aggregate community function such as biomass^3^. The NBES is determined from the difference between observed and expected stability and can be quantified for any metric of stability that is measured by comparing a treatment response to a control (Box 1). Here, we assess the NBES primarily (though not exclusively) using the OEV index, as the OEV integrates the response of the whole system over time and therefore integrates a multitude of stability metrics^23^ (Fig. 1). Calculation of the net biodiversity effect on stability is, however, not limited to any particular stability metric, and can be assessed for any metric that is based on a comparison of a treatment response to a control, such as resistance or temporal variability.

Here, we combine model simulations and a microcosm experiment to determine (i) whether the NBES is consistently positive and (ii) increases with species richness, leading to greater stability in more diverse communities, (iii) how species thermal optima influence the NBES under different temperature regimes, and (iv) how the NBES is influenced by competition strength. We address these questions by quantifying the NBES in model simulations using a temperature-dependent version of the Lotka–Volterra competition model, followed by a microcosm experiment, where we manipulate phytoplankton species richness and temperature disturbance regimes (that is, diurnal temperature fluctuations, a gradual temperature increase, and their combination; see ‘Methods’). We measure community- and species-specific responses to disturbance and determine the NBES for each species combination in both simulations and the experiment. We predict that (i) the net effect of biodiversity on stability is positive (that is, NBES > 0), as the observed stability in species combinations is higher than expected from species in isolation because biodiversity enhances temporal stability^24^; (ii) that NBES increases with increasing species richness, given the positive species richness–ecosystem functioning relationship^22^; and (iii) that NBES varies among species combinations and temperature treatments due to differences in species thermal optima^25–27^. For the simulations, we further expect that the magnitude of the NBES depends on the strength of competitive interactions, given that interactions directly shape species responses to disturbances^28^.

Box 1 Quantification of the net biodiversity effect on stability (NBES) To calculate the net biodiversity effect on stability (NBES), define for any mixture:

**Table:** 

${{{{\rm{M}}}}}_{{{{\rm{Con}}}},{{{\rm{i}}}}},$ ${{{{\rm{M}}}}}_{{{{\rm{Treat}}}},{{{\rm{i}}}}}$	Biomass of species *i* in monoculture under control (*Con*) or treatment conditions (*Treat*) over time.
$\frac{{{{{\rm{M}}}}}_{{{{\rm{Treat}}}},{{{\rm{i}}}}}}{{{{{\rm{M}}}}}_{{{{\rm{Con}}}},{{{\rm{i}}}}}}$	Ratio of biomass dynamics of species *i* in the treatment monoculture $({{{{\rm{M}}}}}_{{{{\rm{Treat}}}},{{{\rm{i}}}}})$ relative to the control $({{{{\rm{M}}}}}_{{{{\rm{Con}}}},{{{\rm{i}}}}})$ over time, indicating the strength of treatment effect.
${{{{\rm{Y}}}}}_{{{{\rm{Con}}}},{{{\rm{i}}}}}$, ${{{{\rm{Y}}}}}_{{{{\rm{Treat}}}},{{{\rm{i}}}}}$	Biomass of species *i* in the multi-species mixture under control (*Con*) or treatment conditions (*Treat*) over time.
${{{{\rm{R}}}}}_{{{{\rm{Con}}}},{{{\rm{i}}}}}$	Relative biomass of species *i* in the control multi-species mixture $({{{{\rm{Y}}}}}_{{{{\rm{Con}}}},{{{\rm{i}}}}})$ over time. Different to the NBEF^22^, we account for temporal dynamics in species relative proportions to separate the effects of disturbance from naturally-occurring community dynamics.
${{{{\rm{N}}}}}_{{{{\rm{Con}}}}}=\,{\sum}_{{{{\rm{i}}}}}{{{{\rm{Y}}}}}_{{{{\rm{Con}}}},{{{\rm{i}}}}}$	Total observed biomass in the control mixture over time (Eq.1)
${{{{\rm{N}}}}}_{{{{\rm{Treat}}}}}=\,{\sum}_{{{{\rm{i}}}}}{{{{\rm{Y}}}}}_{{{{\rm{Treat}}}},{{{\rm{i}}}}}$	Total observed biomass in the treatment mixture over time (Eq.2)
${{{{\rm{E}}}}}_{{{{\rm{i}}}}}={{{{\rm{N}}}}}_{{{{\rm{Con}}}}}\frac{{{{{\rm{M}}}}}_{{{{\rm{Treat}}}},{{{\rm{i}}}}}}{{{{{\rm{M}}}}}_{{{{\rm{Con}}}},{{{\rm{i}}}}}}{{{{\rm{R}}}}}_{{{{\rm{Con}}}},{{{\rm{i}}}}}$	Expected species-specific biomass of species *i* in the multi-species treatment mixture over time (Eq.3)
${{{{\rm{RR}}}}}_{{{{\rm{obs}}}}}=\frac{({{{{\rm{N}}}}}_{{{{\rm{Treat}}}}}-\,{{{{\rm{N}}}}}_{{{{\rm{Con}}}}})}{({{{{\rm{N}}}}}_{{{{\rm{Treat}}}}}+{{{{\rm{N}}}}}_{{{{\rm{Con}}}}})}$	Observed response ratio, calculated for each individual time point (Eq.4)
${{{\rm{RR}}}}_{\exp }=\frac{({\sum}_{{{\rm{i}}}}{{{\rm{E}}}}_{{{\rm{{i}}}}}-{{{\rm{{N}}}}}_{{{\rm{Con}}}})}{({\sum}_{{{\rm{i}}}}{{{\rm{E}}}}_{{{\rm{i}}}}+{{{\rm{{N}}}}}_{{{\rm{Con}}}})}$	Expected response ratio, calculated for each individual time point (Eq.5)

The NBES is then the difference between observed and expected stability (Fig. 1) where, stability is estimated from the observed response ratio $({{{\rm{RR}}}}_{{{\rm{obs}}}}),$ and expected response ratio $({{{\rm{RR}}}}_{\exp })$. We quantified the NBES based primarily on the integrative metric of Overall Ecological Vulnerability (OEV^23^).

While the calculation of the NBES is analogous to that of the net biodiversity effect on functioning^22^, the interpretation of NBES is sign-dependent. If the disturbance at hand decreases biomass, NBES > 0 corresponds to a positive deviation from predictions and a stabilising biodiversity effect (Fig. 1), whereas NBES < 0 indicates a negative deviation from predictions and, thus, a destabilising effect of biodiversity.

## Results

### Model simulations

In our simulation experiment, we assessed the NBES using a temperature-dependent Lotka–Volterra species competition model^29^ with differing levels of competition strength (that is, no competition, intermediate, and strong competition) and different distributions of species-specific thermal optima (all species equal, each species different). The NBES was then assessed for every possible community combination of 2–5 species for three temperature disturbance treatments (increasing temperature from 15 to 20 °C, temperature fluctuations from 15 to 20 °C with a mean temperature of 17.5 °C, and combined temperature increase and fluctuations from 15 to 20 °C with fluctuations of ±2.5 °C, relative to an undisturbed control at 17.5 °C) that were analogous to the temperature disturbance treatments in our microcosm experiment (please see ‘Methods’ for a detailed description of the model).

**Fig. 2:**
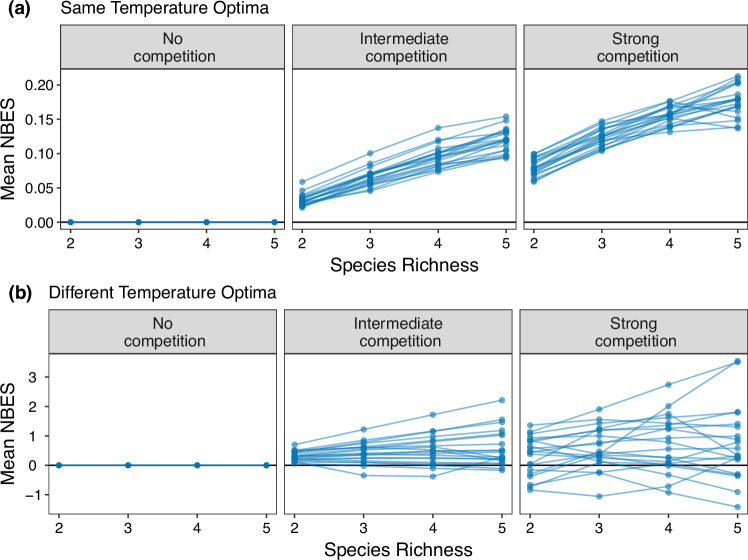
Results of model simulations for gradually increasing temperatures. Mean net biodiversity effect on stability (NBES) as a function of species richness for increasing temperature for model scenarios where (**a**) all species had the same temperature optimum (${{{{\boldsymbol{b}}}}}_{{{{\boldsymbol{opt}}}}}$ = 17.5 °C) and **b** all species had a different temperature optima (${{{{\boldsymbol{b}}}}}_{{{{\boldsymbol{opt}}}}}$ ranging between 15 and 20 °C). We calculated mean NBES across species combinations for each model run separately (*n* = 10 for richness levels 2 and 3, *n* = 5 for richness level 4, and *n* = 1 for richness level 5). Panels indicate increasing competition strength by increasing the standard deviation of α from left to right (α ranged from 0 to 0.5 with 0.5 indicating strong competition). Please note the different scaling of the y-axes in (**a**) and (**b**). Here, we show only the results from the increasing temperature treatment as the results for all three of our disturbance regimes were similar (for a complete overview of NBES in different disturbance regimes, see Supplementary Fig. S2). Source data are provided as a Source data file.

Observed stability differed from expected stability in most of the simulated communities across richness levels. However, the direction of the NBES differed among disturbance types and competition strengths (Supplementary Fig. S1). The strength of competitive interactions modulated the magnitude of the NBES. In simulations that included strong competition, communities exhibited larger variation in NBES, amplifying either stabilising or destabilising biodiversity effects depending on the species trait combination in the model run. When all species had the same temperature optimum of 17.5 °C—near the regime temperature—the NBES was consistently positive and increased with increasing species richness (Fig. 2). In contrast, in model runs where species varied in their temperature optima and with strong competitive interactions, the NBES was more variable and included neutral and negative effects, sometimes resulting in non-monotonic dynamics as richness increased (Fig. 2).

**Fig. 3:**
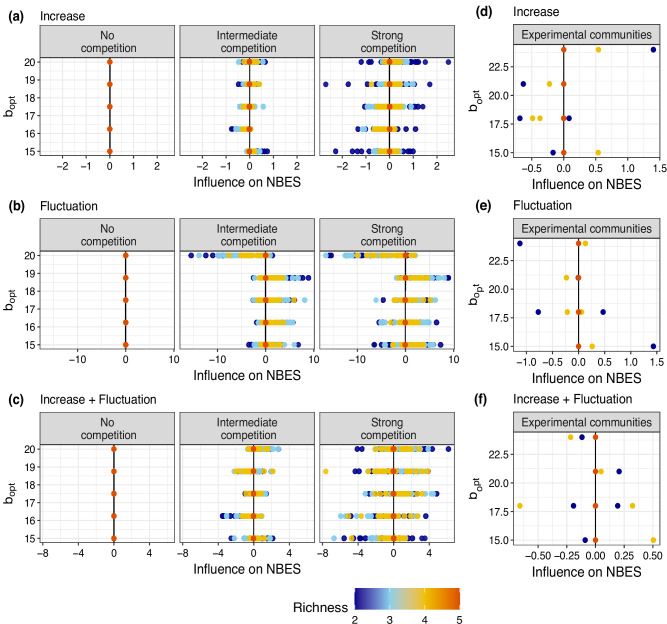
Species influence on NBES as a function of temperature optimum. Relationship between the influence of species on the NBES and their temperature optimum ${{{{\boldsymbol{b}}}}}_{{{{\boldsymbol{opt}}}}}$ for model communities (**a**–**c**) and experimental communities (**d**–**f**). **a**, **d** Increasing temperature, **b**, **e** fluctuating temperatures, and **c**, **f** increasing and fluctuating temperatures. All species had different temperature optima (${{{{\boldsymbol{b}}}}}_{{{{\boldsymbol{opt}}}}}$ ranging between 15 and 20 °C). Each point represents one species. Species influence on NBES is calculated as their mean influence on NBES per richness level for each model run (**a**–**c**) and experimental treatment (**d**–**f**). Panels in (**a**–**c**) indicate increasing competition strength by varying the standard deviation of α from left to right. Species temperature optima (${{{{\boldsymbol{b}}}}}_{{{{\boldsymbol{opt}}}}}$) determined the direction of the NBES. Species richness is indicated by the colour gradient. Please note the different scaling of the x-axes. Source data are provided as a Source data file.

As competition strength increases, species in species-poor communities contributed more strongly to NBES than those in species-rich assemblages (Fig. 3), while their temperature optima (${{b}}_{{opt}}$) determined the direction of the effect. Specifically, species with temperature optima at the extremes of the simulated range showed the greatest positive or negative influence on NBES under increasing temperatures. Under fluctuating temperatures, however, species with the highest thermal optima influenced the NBES negatively, whereas species with lower thermal optima showed mostly positive influences on NBES. The combined treatment mirrored the effects found for increasing temperatures but with greater variation in the influence on NBES for strong competition. In general, stronger competition resulted in more variable influences on NBES, reinforcing the role of interactions in shaping stability outcomes. Overall, these results demonstrate that biodiversity effects on stability are not uniform, but are mediated by species interactions and temperature-dependent species performance.

### Microcosm experiment

In our microcosm experiment, we used five diatom species isolated from the North Sea and manipulated all possible species combinations across a gradient of richness (one, two, four, and five species). Cultures were exposed to one of four temperature treatments comprising a gradual temperature increase of 0.2 °C per day, diurnal temperature fluctuations of ±3 °C, the combination of gradual increase and fluctuations, and a constant temperature control, all starting at 17 °C or with a mean of 17 °C (see ‘Methods’).

**Fig. 4:**
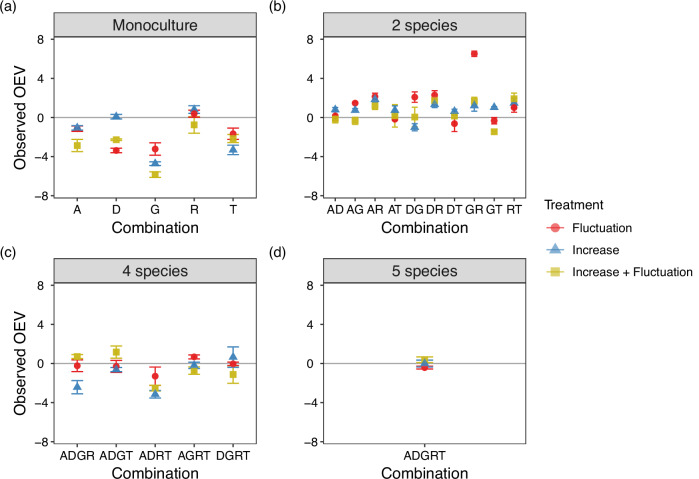
Observed instability, measured as overall ecological vulnerability (OEV) for different species combinations. Mean (*n* = 3 biological replicates/microcosms) observed instability ± SE for (**a**) monocultures, **b** two-species assemblages, **c** four-species assemblages, and **d** five-species assemblages. Higher absolute values indicate greater instability that can result from positive and negative deviations in the treatment relative to the control. Different colours and shapes indicate different temperature treatments: diurnal fluctuations ±3 °C with a mean of 17 °C (red circles), temperature increase from 17 to 23 °C (blue triangles), and combined diurnal temperature fluctuation of ±3 °C around an increasing mean (yellow squares). Phytoplankton species are abbreviated as A—*Asterionellopsis*, D—*Ditylum*, G—*Guinardia*, R—*Rhizosolenia*, T—*Thalassionema*. Source data are provided as a Source data file.

Observed stability showed considerable variation across treatments and richness levels as well as associated species combinations (Fig. 4). Most species monocultures showed negative deviations for disturbances involving fluctuations, whereas gradual temperature increases led to positive deviations in some monocultures (Fig. 4). The majority of two-species combinations showed positive or near-neutral deviations from the constant temperature controls. In contrast, four-species combinations deviated both positively and negatively from their controls, with outcomes driven largely by which species were present rather than by richness itself.

**Fig. 5:**
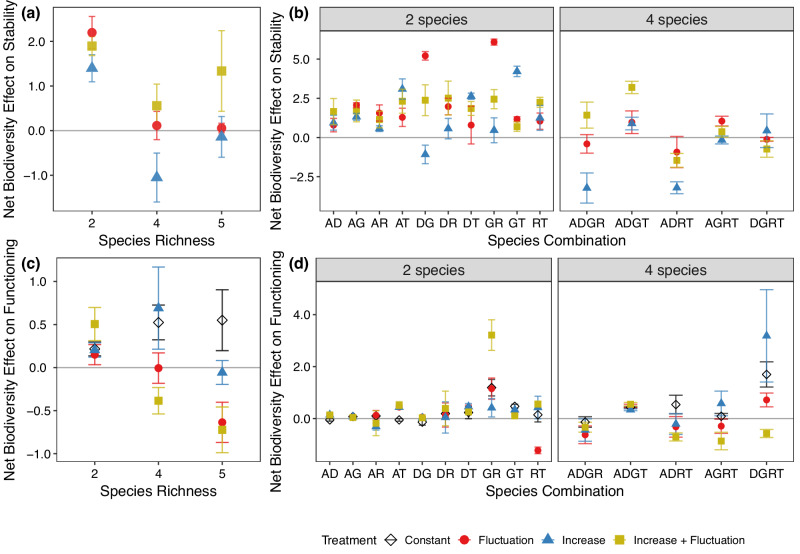
Net biodiversity effects on stability and ecosystem functioning. The net biodiversity effect on stability (NBES) for the different (**a**) species richness levels and **b** species combinations, and the net biodiversity effect on ecosystem functioning for different (**c**) species richness levels and **d** species combinations. Positive values of the net biodiversity effect on functioning and stability indicate a positive effect of biodiversity, and negative values a negative effect. Different colours and shapes indicate different temperature treatments: diurnal fluctuations ±3 °C with a mean of 17 °C (red circles), temperature increase from 17 to 23 °C (blue triangles), and combined diurnal temperature fluctuation of ±3 °C around an increasing mean (yellow squares). Phytoplankton species are abbreviated as A—*Asterionellopsis*, D—*Ditylum*, G—*Guinardia*, R—*Rhizosolenia*, T—*Thalassionema*. Each point indicates mean ± SE with *n* = 3 for plots (**b**), (**d**) and varying replicates for (**a**) and (**c**), where *n* = 30 for two species combinations, *n* = 15 for four-species combinations, and *n* = 3 for five-species combinations. Source data are provided as a Source data file.

Observed stability differed from expected stability in most communities of two, four or five phytoplankton species (Fig. 5a). The NBES was overall positive and differed significantly from zero (*t*-test across all compositions; df = 143, *p* < 0.0001, *t* = 7.0697, 95% CI [0.81, 1.45]). Moreover, both temperature treatment (LM, df = 135, *p* = 0.0115) and species richness (LM, df = 135, *p* < 0.0001) had a significant effect on NBES whereas their interaction was not significant (LM, df =135, *p* = 0.5197) (Supplementary Tables S1 and S3; Fig. 5b). Specifically, for the combined temperature increase and fluctuations treatment the NBES was positive or neutral overall and decreased for four- and five-species assemblages (Fig. 5a). For the increasing temperature treatments, the NBES even turned negative at high richness levels. NBES of both four- and five-species assemblages differed significantly from those of two-species combinations (planned pairwise-comparisons two species vs 5 species, *p* = 0.0135; planned pairwise-comparisons two species vs 4 species, *p* < 0.0001; Supplementary Table S2), but not from each other (planned pairwise-comparisons 4 species vs 5 species, *p* = 0.3859; Supplementary Table S2). Disentangling species effects on NBES revealed large differences among disturbance regimes. That is, species influence on the NBES aligned with their temperature optima (Fig. 3). Under gradually increasing temperatures, species with high temperature optima (24 °C) positively impacted NBES, while a gradual increase in temperature led to suboptimal conditions for species with lower temperature optima, thereby negatively affecting NBES (Fig. 3d). Under fluctuating disturbances, species with low temperature optima had a positive influence on NBES, whereas those with higher temperature optima contributed mostly negatively (Fig. 3e). Combined increasing temperature and fluctuation mirrored the patterns found for fluctuating temperatures but with larger variation (Fig. 3f). Overall, species in two-species communities exerted a stronger influence on NBES compared to those in multi-species assemblages of four or five species.

The net biodiversity effect on functioning was overall positive (*t*-test across all compositions; df = 192, *p* < 0.0001, *t* = 3.4949, 95% CI [0.09544018, 0.34274907]), but varied significantly with species richness and temperature treatments in interaction (LM, df = 180, *p* = 0.0048, Supplementary Table S1). Specifically, the net biodiversity effect on functioning was always positive in the constant temperature control and increased with increasing species richness (Fig. 5c). In contrast, disturbance treatments decreased the net biodiversity effect on functioning as species richness increased, and even turned it negative in most cases for four- and five-species combinations (Fig. 5d). One exception was the gradual temperature increase with a positive net biodiversity effect on functioning for four-species assemblages due to an increase in total biomass at the end of the experiment driven by *Thalassionema* (Supplementary Figs. S2, S3 and S4). Overall, the net effects of biodiversity on stability and functioning correlated positively (Spearman-Rank correlation, *R* = 0.35, *p* < 0.0001).

## Discussion

Community-wide functional stability could not be predicted by species-specific responses to the same environmental change in monocultures. Rather, simulated communities showed both lower and greater stability than expected, whereas experimental communities showed overall greater stability than expected (that is, an overall positive NBES). NBES varied widely in direction and magnitude in the simulations, while in the experiment NBES was highest for two-species combinations and decreased at higher species richness levels. NBES varied among species combinations and temperature treatments according to species thermal optima in both simulations and the experiment, and even became negative for some. Further, the simulations highlighted the combined role played by temperature optima and species interactions for NBES. Specifically, the magnitude of the NBES varied with the strength of competition in our simulated model communities. Our approach enabled us to quantify the amount of additional stability derived from biodiversity, in terms of both richness and composition, therefore providing important insights into the biodiversity–stability relationship.

We first tested the NBES approach using simulated data with varying competition strengths, disturbance regimes, and distributions of thermal optima. Our framework enabled consistent estimation of NBES across all modelled conditions, including cases with minimal or neutral biodiversity effects. The direction and magnitude of the NBES depended on competition strength and species’ temperature optima. When species shared a favourable optimum, that is all species had their temperature optimum at the mean temperature of temperature fluctuations (17.5 °C), NBES was consistently positive and increased with increasing competition strength. In contrast, greater variation in species thermal optima led to more variable NBES outcomes with much greater magnitude. This suggests that the effect of biodiversity on stability depends on how well the traits of a community’s component species match the prevailing environmental conditions^30,31^. That is, how well the temperature optima match the regime temperature. In the absence of thermal differentiation, stronger competition enhanced differences in species’ biomass contributions, such that highly competitive species achieved higher biomass than expected, thereby elevating overall stability through increased mean performance^6,32,33^. In contrast, when species differed in their thermal optima, NBES became more variable and included neutral or negative effects, particularly under strong competitive interactions (Fig. 2). This variability may have arisen because differences in (thermal) traits have the potential to alter competitive hierarchies and reduce synchrony in population responses^24^, leading to both stabilising and destabilising outcomes depending on how species’ traits align with the prevailing temperature regime. In some cases, strong competition and high diversity in species temperature optima led to non-monotonic relationships between richness and biodiversity effects on stability (Fig. 3 and Supplementary Fig. S1). This was more often the case for our temperature increase treatment than for temperature fluctuations. In the former, species with stronger competitiveness showed greater variation in their influence on the NBES, as interactions tend to modulate species responses to disturbance^28^. It has recently been suggested that species dynamics are more important drivers of biodiversity effects on stability than interspecific interactions^19^. Our results highlight that both processes act jointly. Specifically, competition strength and the distribution of species’ thermal traits jointly determine the direction and variability of biodiversity effects on stability, with high competition and high trait differentiation promoting variable, context-dependent outcomes^17,32^.

We also find greater variation in the direction of the NBES with increasing species richness. This suggests that more diverse communities will exhibit a wider range of stability outcomes. This is consistent with the theoretical work of Ives and Carpenter^11^, who showed that species-rich systems are more likely to show a broader range of diversity–stability relationships in the face of disturbance because interspecific interactions can both amplify and dampen stability effects.

In our microcosm experiment, the NBES was overall positive, which we expected based on previous reports that biodiversity dampens the variability of emergent properties of the community in fluctuating environments^5,6,9,34^. Contrary to expectations that the NBES would increase with increasing species richness, however, we found that the mean NBES decreased for higher levels of species richness (Fig. 5). One reason for this may be the lower resistance of species-rich assemblages to disturbance treatments than expected from monoculture and compared to two-species assemblages, resulting in a negative trend for the NBES for resistance along the species richness gradient (Supplementary Fig. S5). If species-rich systems have a greater chance of harbouring species that grow well under undisturbed conditions, as suggested by the biodiversity insurance hypothesis^13^, they also have a greater chance of losing this growth potential under disturbance. This is especially true if there exists a trade-off between species growth and resistance to disturbances^10,14^. Such trade-offs can reshuffle competitive hierarchies during disturbance events, reducing the contribution of species that would otherwise dominate under benign conditions^10,14,35^ and thereby suppressing NBES at higher richness. At the same time, this investment in growth rather than resistance in species-rich communities may allow for compensatory fluctuations due to asynchronous responses among species over time^6,24,36^ despite the sustained disturbance pressure. Consistent with this, we also found lower NBES for temporal variability in species-rich assemblages than in two-species assemblages for the increasing temperature regime (Supplementary Fig. S5), indicating that observed variability was lower than expected. This suggests a negative correlation between resistance and temporal stability for increasing temperature regimes. Consistent with this, Pennekamp et al.^8^ also reported negatively covarying stability metrics under a temperature gradient of constant temperatures (that is, high temporal stability and low resistance), which resulted in a ‘U’-shaped relationship between overall ecosystem stability and species richness. For disturbances involving fluctuations, we found higher temporal variability with increasing species richness, likely reflecting short-term environmentally driven responses to fluctuating temperatures, where differences in species’ thermal optima lead to shifting dominance^35^, but limited compensatory dynamics^18,24^, thereby increasing community-level variability. Indeed, a study on plankton communities showed that environmental fluctuations can disrupt compensatory dynamics and increase short-term variability in community biomass^36^. Combined, this highlights the necessity of considering the multidimensional nature of stability^2,37^.

The net biodiversity effect on functioning largely mirrored those for stability in our microcosms, decreasing with increasing species richness when exposed to disturbance treatments, but increasing in the constant temperature controls of our experiment. The positive relationship between productivity and species richness, as in our controls, is a frequent observation in diversity manipulations and often attributed to the fact that, in more diverse ecosystems different species use resources in different ways, leading to more efficient resource utilisation^10,13,19,38^. Most studies in the context of biodiversity and ecosystem functioning have been conducted in undisturbed systems^38,39^, which may not fully capture the dynamics under varying environmental conditions. However, disturbances have the potential to disrupt this biodiversity–productivity relationship, and even turn it negative, due to the trade-off between resistance to disturbance and growth rate^10^. This context-dependency of the net biodiversity effect on functioning in our experiment suggests a positive correlation between productivity and stability in our experimental system.

Overall, both simulations and experiments revealed high heterogeneity in the NBES within richness levels and among species combinations, reflecting diverse community responses to disturbance regimes. Much of this variation was driven by species’ thermal optima—species whose traits matched the prevailing temperature regime tended to have a more positive effect on the NBES (Fig. 3). This variation in the influence of different species on the NBES based on their thermal optima suggests a strong idiosyncratic compositional effect. That is, rather than having a uniform response across the entire community, individual species may exhibit distinct responses to the different temperature regimes and, therefore, affect stability in different ways in different contexts^40–43^. Such variation aligns with known differences in species’ thermal responses and metabolic rates^25,26,35^. For example, gradual warming often reduces growth^27^, as shown in *Asterionellopsis, Guinardia*, and *Thalassionema* monocultures, while *Ditylum* and *Rhizosolenia* performed better (Supplementary Fig. S2). Diurnal fluctuations led to lower biomass in most species, consistent with predictions from non-linear averaging^44^. While temperature fluctuations around a gradually increasing mean temperature represented a brief exposure to temperatures close to the thermal optimum for many species^45^, this resulted in higher biomass production than under ‘simple’ diurnal fluctuations. These differences in species responses may also reflect different adaptation mechanisms of species, such as reductions in cell size and increased nutrient storage^41,46^, or temporal differences in these adaptation mechanisms^47,48^.

Disturbance regimes might also affect the outcome of interspecific interactions differently. Rising temperatures may lead to increased diversification of species interactions in communities because of buffering mechanisms to environmental change^24,42^. Specifically, rising temperatures may enhance competition between species due to the critical role of metabolic traits in determining the impact of temperature changes on interspecific interactions^35^. Such increased competition may have manifested in a negative NBES for higher levels of species richness in our experiment, which is consistent with results from the model simulations for strong competitive interactions (Fig. 2). In contrast, temperature fluctuations may increase the likelihood of long-term species asynchrony, thereby facilitating community and ecosystem stability, despite potential short-term destabilising effects^13,24,36^. This is consistent with our results of the experiment and model simulations for disturbances involving fluctuations, where we see greater variation in species contributions to NBES based on their thermal optima.

We applied the NBES approach to simulated multi-species communities and experimentally to microcosm communities with undisturbed controls. Our approach could, however, also be applied to observational data if post-disturbance dynamics of both mono-specific and species-specific responses in the community are available. Specifically, if observational time series data include both monoculture dynamics and species-level contributions within mixed communities, NBES can be calculated by comparing observed community stability against expectations derived from single-species responses, enabling application of the framework across different ecosystems and disturbance scenarios.

Given the multitude of experimental contexts and different outcomes in combination with model simulations using Lotka–Volterra dynamics, our experimental design was ideal for testing the difference between expected and observed stability. Our simulations cover a range of competition strengths and distribution of thermal optima, showing that the NBES can be both positive and negative, depending on community and trait composition. However, our microcosm experiment was based on a single system using highly productive marine primary producers. We therefore encourage further studies to determine the NBES for other organism groups in other systems to explore potential trade-offs between both stability and productivity^10,17,49^, and the importance of interaction type (e.g., competition vs. mutualism).

The various effects of different disturbance treatments on the net effect of biodiversity on stability and functioning highlight the need to consider a wide range of disturbance types and characteristics when studying the consequences of biodiversity. Given the scale-dependency of biodiversity^50^, it is essential to broaden the spatial and temporal scope of biodiversity research beyond individual systems (e.g., by using a food-web design) and specific time frames. In particular, the introduction of a greater number of species and species richness levels could further elucidate the dynamics of multi-species assemblages. This will facilitate a deeper understanding of the underlying mechanisms that govern the biodiversity–stability relationship and how they may change over space and time.

In summary, by quantifying the additional stability provided by biodiversity under three temperature change scenarios using both model simulations and a microcosm experiment, we show that stability emerges as a community property that is influenced not only by species richness but also by species identity. Different species played key roles in determining community stability under different disturbance regimes depending on their thermal optima. This suggests that species loss may have unforeseen and severe consequences for ecosystems under climate change and highlights the context-dependency of biodiversity effects on stability. In practical terms, our findings highlight the importance of maintaining biodiversity to enhance ecosystem resilience to external forcing and maintain productivity, and thus provide crucial insights for managing ecosystems in times of biodiversity crisis and changing environmental conditions.

## Methods

### Model simulations

We simulated a five-species community using a continuous Lotka–Volterra model with temperature-dependent growth rates^29^. We used a continuous temperature-dependent Lotka–Volterra model (instead of a time-discrete model^51^) to capture the time-varying effects of temperature on population dynamics. In the model, the change of biomass for each species is described as

6 $$\frac{{d}{{{N}}}_{{ i}}}{{dt}}({{{\rm{T}}}})={{r}}_{{i}}({{{\rm{T}}}})\,{{N}}_{{i}}\left({{1}}-\frac{{\sum }_{{ j}={{1}}}^{{{5}}}{{{\alpha }}}_{{i},{j}}{{N}}_{{j}}}{{{K}}_{{i}}({{{\rm{T}}}})}\right)$$

where ${{{N}}}_{{{ i}}}$ corresponds to the species-specific biomass, *r*_*i*_(*T*) is the species-specific temperature-dependent intrinsic growth rate, which is given by the difference between the birth rate $\left({{{ b}}}_{{{0}},{{ i}}}({{ T}})\right)$ and death rate $({{{ d}}}_{{{0}},{{ i}}}({{T}}))$, ${{{ K}}}_{{{ i}}}({{ T}})$ the species-specific temperature-dependent carrying capacity, ${{{\alpha }}}_{{{ i}},{{ j}}}$ the competition strength between species *i* and species *j*, and ${{{ N}}}_{{{ j}}}$ the biomass of species *j*.

The carrying capacity *K*_*i*_(*T*) of species *i* is given as

7 $${{{ K}}}_{{{ i}}}({{{\rm{T}}}})=\frac{{{{ r}}}_{{{ i}}}({{ T}})}{{{\beta }}+{{\delta }}}\,$$

where *β* and *δ* are the density dependent constants that were both set to 0.025.

Temperature dependence (*T*) was incorporated into the birth and death rate:

8 $${{{ b}}}_{{{{\bf{0}}}},{{ i}}}\left({{ T}}\right)={{{ a}}}_{{{ b}}}{{{ e}}}^{\frac{{-\left({{ T}}-{{{ b}}}_{{{opt}},{{ i}}}\right)}^{{{2}}}}{{{{ s}}}_{{{ i}}}}}$$

9 $${{{ d}}}_{{{0}}}\left({{ T}}\right)={{{ a}}}_{{{ d}}}{{{ e}}}^{{{zT}}}$$

where ${{{ a}}}_{{{ b}}}$ and ${{{ a}}}_{{{ d}}}$ are the intercepts of birth and death rates, respectively, ${{{ b}}}_{{{opt}},{{ i}}}$ is the species-specific temperature at which the intrinsic growth rate is highest, ${{ s}}$ scales the width of the temperature response of birth rate, ${{ z}}$ is the slope of the death rate and scales the effect of temperature (in °C) to mimic the Arrhenius relationship^29^. Parameter values were set so that the community would reach equilibrium biomass within 150-time steps (see Supplementary Table S4 for an overview of introduced parameter values). We assumed intraspecific competition in communities to be stronger than interspecific competition. This is incorporated by setting the competition terms ${{{\alpha }}}_{{{ i}},{{ j}}}$ along the diagonal of the competition matrix to 1, which is the maximum value of terms ${{{\alpha }}}_{{{ i}},{{ j}}}{{{\boldsymbol{.}}}}$ The interspecific competition terms ${{{\alpha }}}_{{{ i}},{{ j}}}$ were drawn from a one-sided normal distribution *α* > 0, so that all interactions were competitive, with asymmetric interactions between any two species in the model. A higher value for α indicates a stronger effect by species *i* on species *j*, whereas a low value of *α* indicates a small effect of species *i* on species *j*. We varied interspecific competition terms by introducing differing standard deviations (SD) of competition values *α*_*i,j*_ while the mean remained constant (*µ* = 0). Specifically, we tested three competition strengths in the communities:

(i) no competition (SD = 0);
(ii) intermediate competition strength (SD = 0.25); and
(iii) high competition strength (SD = 0.5).

The distribution of species’ temperature optima (${{{ b}}}_{{{opt}},{{ i}}}$) within communities was evenly distributed along specific temperature gradients to represent varying levels of diversity in species responses. We specifically simulated the following two scenarios:

(i) All species had the same ${{{ b}}}_{{{opt}},{{ i}}}$ (17.5 °C), simulating a homogenous response; and
(ii) All species had different ${{{ b}}}_{{{opt}},{{ i}}}$, that were evenly distributed between 15 °C and 20 °C.

We simulated species within each community as monocultures and in every possible combination of 2–5 species. Each of the species assemblages was then exposed to a disturbance and a control run of 150-time steps. The disturbance regimes comprised of a temperature increase from 15 to 20 °C, temperature fluctuations from 15 to 20 °C within one time step with a mean temperature of 17.5 °C (fluctuations), a combination of temperature increase from 15 to 20 °C with fluctuations of ±2.5 °C, and an undisturbed control at 17.5 °C constant. The temperature scenarios commenced at the first time step to be consistent with our microcosm experiment (see below). To account for the stochasticity when drawing the competition values *α*_*i,j*_, each combination of disturbance type (press, fluctuations, combination), competition strength, and temperature optima distribution (${{{ b}}}_{{{opt}},{{ i}}}$) was repeated 20 times, resulting in a total of 480 scenarios (see Supplementary Fig. S6 for an exemplary model run). All simulations were done in R version 4.4.3^52^ using packages here^53^, ggbeeswarm^54^, foreach^55^, svMisc^56^, parallel^52^, and tidyverse^57^.

### Microcosm experiment

**Fig. 6:**
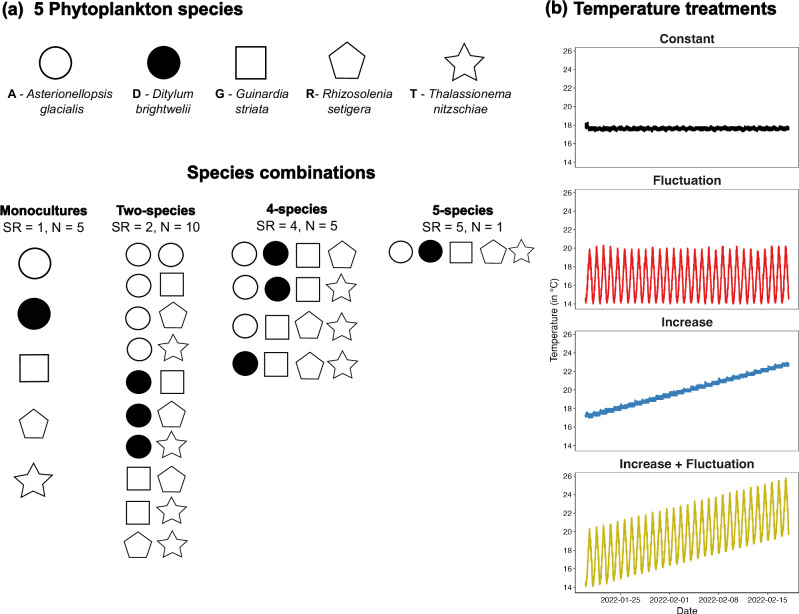
Overview of our microcosm experimental design. Our microcosm experiment comprised (**a**) four levels of species richness, comprising monocultures of each respective species, two species in combination, four species in combination and all five species in combination crossed with (**b**) four temperature treatments, with each temperature treatment-species combination replicated three times. Each shape represents one species. Displayed temperature curves are measured temperatures for the different treatments over the experimental period for one replicate mesocosm each. All temperature treatments started at 17 °C or with a mean temperature of 17 °C and consisted of a constant temperature treatment with 17 °C, a diurnal temperature fluctuation ±3 °C from 14 to 21 °C, a fluctuation combined with increasing temperature where the temperature fluctuated ± 3 °C around the increasing mean, and a temperature increase from 17 to 23 °C.

To quantify the NBES empirically in real communities, we conducted a microcosm experiment on marine phytoplankton with different levels of species richness, from monocultures to multi-species assemblages of five species, and crossed that gradient with four temperature treatments (Fig. 6). Phytoplankton communities are an ideal system to study the effects of biodiversity on stability to disturbance because of their short generation times and high sensitivity to environmental changes^3,58,59^, while supporting high species diversity^60^. We used five different phytoplankton species isolated from the North Sea in summer 2017 that differed considerably in cell volume: *Asterionellopsis glacialis* (252 µm^3^), *Ditylum brightwelii* (2204 µm^3^), *Guinardia striata* (5667 µm^3^), *Thalassionema nitzschioides* (7236 µm^3^), and *Rhizosolenia setigera* (205,215 µm^3^). Mean cell volume was estimated from 20 individuals of each diatom species at the beginning of the experiment under an inverted microscope. We chose these species because of their large variation in cell size, which is known to be a master trait in phytoplankton, influencing differences in growth rates, responses to temperature, and nutrient prevalence^61–63^. In addition, diatoms play a key role in marine primary production worldwide^64^.

Prior to the start of the experiment, cultures were grown at 12 °C in F2 medium at 25 PSU at ~200 µmol s^−1^ in a 12:12 dark:night cycle. To create disturbance regimes with negative effects on species growth rates, each temperature treatment started at 17 °C or with a mean temperature of 17 °C and reached maximum temperatures of 23 °C, thereby exceeding (or coming close to) the average temperature optimum of marine phytoplankton species from the North Sea at 18 °C^45^. In our experiment, species temperature optima (assessed as maximum biomass along a temperature gradient) ranged from 15 to 24 °C with 18 °C for *Asterionellopsis*, 21 °C for *Ditylum*, 15 °C for *Guinardia*, 18 °C for *Rhizosolenia*, and 24 °C for *Thalassionema*. we introduced four different temperature treatments (Fig. 6): a constant temperature control at 17 °C, a diurnal fluctuation treatment from 14 to 20 °C in a 12:12 cycle, a warming treatment with temperatures increasing by 0.2 °C per day from 17 °C to 23 °C, and diurnal temperature fluctuations combined with warming with a fluctuation of ±3 °C around an increasing mean of 0.2 °C per day. We chose a gradual increase in temperature and temperature fluctuations as disturbance treatments on the one hand because pulse disturbances have received considerably more attention than other forms of disturbance in stability research^11,65^. On the other hand, diurnal temperature fluctuations and fluctuations around an increasing mean represent more realistic disturbances compared to pure press or pulse disturbances^37^ and compared with other fluctuation frequencies^66^.

Our experiment comprised four levels of species richness (Fig. 6), each of which was replicated three times. Species richness levels comprised monocultures of each of our five focal species—two species in combination, four species in combination, and all five species in combination. We estimated the density of our pre-cultures photometrically and added a fixed fraction to the medium to initiate all mono- and multi-species cultures at an optical density of ~0.05. Monocultures and multi-species communities (containing two to five species) were assembled using a substitution design. All cultures were grown in a total of 35 ml medium and in sterile 50 ml polyethylene cell culture flasks (Sarstedt).

The experiment was conducted in the ‘Planktotrons’ indoor mesocosm facility^67^, custom-tailored for plankton experiments at the Institute for Chemistry and Biology of the Marine Environment (ICBM). Temperature regimes were created using water baths, thereby controlling the temperatures with the heating and cooling units of the Planktotrons (Fig. 6). The experiment lasted 30 days and all treatments started at day 0 on 19 January 2022. The samples were kept in a 12:12 light:dark cycle at ~150 µmol s^−1^. Nutrient concentrations were chosen to be close to ambient conditions (that is, measured dissolved nutrient concentrations were N 18 µmol, Si 17 µmol, P 1.5 µmol on day 0).

For sampling, experimental units were taken out of the water bath, stirred gently to homogenise the culture and a sample of 0.5 ml transferred into 48-well plates (Sarstedt). We sampled each experimental unit every sixth day and fixed samples with 50 µl of 10% Lugol’s iodine, resulting in a total of six time points over 30 days (that is, days 0, 6, 12, 18, 24, 30). We derived the biomass of individual species by measuring their density under an inverted microscope at 200–400x magnification and estimating their biovolume^68,69^.

### Net biodiversity effect on stability calculation based on different stability metrics

The net biodiversity effect on stability (NBES) can be quantified as the difference between observed and expected stability in experimental communities. While observed stability is determined based on community responses to disturbance in the treatment relative to an undisturbed control, monospecific responses to disturbance in comparison to an equivalent undisturbed control are used to calculate an expected community response. This expected stability derives from the fundamental null assumption that each species shows proportionally the same response to the disturbance in monoculture and mixture, which we can sum according to species’ initial relative abundance in the assemblage.

In this study, we assess the NBES primarily (though not exclusively) using the OEV index, as it integrates the systems response over time and therefore encorporates a multitude of stability metrics^23^. Calculation of the net biodiversity effect on stability is, however, not limited to any particular stability metric, and can be assessed for any metric that is based on a comparison of a treatment response to a control, such as resistance or temporal variability (see below). The calculation of stability should, however, be based on standardised response ratios (RRs) instead of log-response ratios (LRRs), to allow for the possibility that species become locally extinct, which would result in an undefined LRR. The standardised response ratio (RR) thereby is calculated from the difference in community/population biomass in a disturbed setting relative to an undisturbed (control) setting. By comparing the dynamics of a disturbed community with those of an undisturbed control community, it is possible to account for natural variations in biomass that are unrelated to the applied disturbance, and thereby to isolate the difference attributable to the treatment itself. Because instability is defined as the difference between disturbance and control trajectories, it is not meaningful to further compare this quantity to fluctuations occurring within the control community alone.

In contrast to the net biodiversity effect on functioning^22^, quantification of NBES relies on relative rather than absolute responses to an undisturbed control. It does not, therefore, allow the decomposition into selection and complementarity effects, which are based on absolute species responses^22^ (but see ref. ^70^ for a complementary framework that decomposes biodiversity effects on resistance and resilience, without reference to an undisturbed control). By relating biodiversity effects to how species traits align with environmental conditions, the NBES instead offers a powerful means to disentangle the effects of disturbance regimes on community dynamics and to link these to species traits, such as, for example, their thermal optima (see ‘Results’).

### Stability metrics

The OEV instability metric is calculated as the area under the curve of the total biomass in disturbed treatments relative to the undisturbed control. The OEV encompasses several dimensions of post-disturbance-stability^23^ (cf. Supplementary Fig. S7) and directly correlates with the effect size of the disturbance: A larger OEV indicates greater temporal variability, lower resistance to disturbance, slower resilience (recovery rate) and/or incomplete recovery^2,23^. Here, we allow negative and positive values of the area under the curve, thus allowing both negative and positive deviations from controls to cancel^51^. We then calculate NBES as the difference between observed OEV and expected OEV (Box 1), assessed as the difference in the area under the curve (AUC) of the expected standardised response ratios (RR) (1) and observed standardised RR (2), respectively, over time, that is, as:

10 $${{N}}{{{BES}}}_{{{OEV}}}={{AUC}}({{RR}}_{{obs},{{ t}}})-{{{AUC}}}({{RR}}_{{{\exp }},{{{\bf{t}}}}})$$

A positive NBES then indicates a stabilising effect of biodiversity, as measured instability is lower (less negative) than expected (that is, observed OEV – expected OEV is positive).

For data from the microcosm experiment, we also assessed the NBES for both resistance and temporal variability. Depending on the stability metric of interest, calculations of NBES are based on response ratios over time as for OEV or temporal stability or are limited to a specific time point as for resistance.

The NBES for resistance is calculated as the difference between observed and expected RR at the first sampling (*t*_1_) after the commencement of disturbance, that is on day 6:

11 $$N{{BES}}_{{resist}}=\,{{RR}}_{{obs}}\left({t}_{1}\right)-{{RR}}_{\exp }\left({t}_{1}\right)$$

The NBES for temporal variability is determined from the difference in the coefficient of variation (CV) from observed and expected absolute ${{RR}}$ over time:

12 $${{NBES}}_{{CV}}={{CV}}( {|} {RR}_{{obs},{t}} {|} )-{{CV}}( {|} {RR}_{\exp,{{{\rm{t}}}}} {|} )$$

13 $${{{\rm{with}}}}\,{{CV}}=\frac{{{SD}}}{{{Mean}}}$$

### Data analyses

All analyses were conducted using the R programming language in R version 4.5.3^71^. For calculation of the area under the curve, we applied the auc() function within the MESS package^72^ using linear splines and allowing for negative areas. For analysis we used packages available within the tidyverse^57^, ggpubr^73^, patchwork^74^, cowplot^75^, and here^53^. Conceptual figures were created using Inkscape 1.2 (version dc2aeda, 2022-05-15).

To test for a species identity effect of individual species on the NBES, we analysed the relationship between species temperature optima and the influence on NBES. Specifically, we calculated the species-specific influence on NBES as $\bar{{{ X}}}-\bar{{{ A}}}$, where $\bar{{{ X}}}$ represents the grand mean over all species combinations per richness level and $\bar{{{ A}}}$ is the species-specific mean over all combinations that include species *A*. A species had a positive effect on the NBES when the NBES in combinations that included the specific species ($\bar{{{ A}}}$) was greater than the average NBES ($\bar{{{ X}}}$).

To test for the effect of temperature treatments and species richness on NBES in experimental communities, we conducted linear models with species richness and temperature treatment as fixed effects. Additionally, we conducted individual planned comparisons to compare the difference in NBES across species richness levels within treatments. Specifically, we performed a planned contrast by subsetting the data to these richness levels and defining a two-level factor (two vs. four species, two vs. five species, and four vs. five species). We then fitted a linear model including temperature treatment, richness contrast, and their interaction using a two-sided analysis of variance. Species richness levels were treated as factors here to account for the differences in observation numbers.

The net biodiversity effect on functioning was calculated following Loreau and Hector (2001)^22^ from the observed and predicted biomass yield at the end of the experiment (*t*_max_). Similar to the NBES, we tested for an effect of temperature treatments and species combinations on the net biodiversity effect on functioning using linear models with species richness and temperature treatment as fixed effects.

### Reporting summary

Further information on research design is available in the Nature Portfolio Reporting Summary linked to this article.

## Supplementary information

Supplementary Information

Reporting Summary

Transparent Peer Review file

## Source data

Source Data

## Data Availability

The experimental data, including all raw data, generated in this study have been deposited in the Figshare database under accession code 10.6084/m9.figshare.25568490.v3^76^. The simulated data generated in this study have been deposited in the Zenodo database under accession code: 10.5281/zenodo.15274625^77^. Source data are provided with this paper.

## Code availability

All code used for the analysis of the experimental data is permanently archived on Zenodo 10.5281/zenodo.19495106^78^. Code for the simulations is permanently archived on Zenodo under the accession code: 10.5281/zenodo.19495091^79^.
