# Supplementary material for: Quantifying the net effect of biodiversity on ecological stability
Source: Nat Commun. 2026 Jul 7;17:5942. doi: 10.1038/s41467-026-75047-z (PMC13341745; doi:10.1038/s41467-026-75047-z)
Supplement: Supplementary file 1 — Supplementary Information [file 41467_2026_75047_MOESM1_ESM.pdf]

Supporting Information

Quantifying the net effect of biodiversity on ecological stability

Authors: Charlotte Kunze*, Dominik Bahlburg, Maren Striebel, Toni Schott, Ian Donohue & Helmut Hillebrand

*corresponding author: charlotte.kunze@hifmb.de

Supplementary Table S1. The effect of species richness and temperature treatments on Net Biodiversity Effects. Results of the linear models for the net biodiversity effect (NBE) on Stability (df = 135) and the NBE on Functioning (df = 180) with species richness and temperature treatments (Temp) as fixed effects. Significant effects are indicated in bold.

**Variable**	**NBE on Stability**			**NBE on Functioning**		
	**numDF**	**F-value**	**p-value**	**numDF**	**F-value**	**p-value**
Temp	2	4.61255	**0.0115**	3	1.387293	0.2482
Richness	2	21.6923 6	**<0.0001**	2	1.860254	0.1586
Temp: Richness	4	0.81175	0.5197	6	3.237240	**0.0048**

Supplementary Table S2. Results of the planned pair-wise comparisons between richness levels. Pairwise comparisons were performed using separate linear models for each richness contrast that is two vs 4, two vs 5, and 4 vs 5 species. P-values were Tukey-adjusted within each set of comparisons. Degrees of freedom differ because each contrast was fitted using a separate model. Significant effects are indicated in bold.

**Contrast**	Estimate	**SE**	**df**	**t.ratio**	**p.value**
**2 – 4**	**1.96**	**0.308**	**129**	**6.360**	**<0.0001**
**2 – 5**	**1.41**	**0.561**	**93**	**2.517**	**0.0135**
4 – 5	-0.544	0.622	48	-0.875	0.3859

Supplementary Table S3. Estimated marginal means from the linear models. Results of the postHoc analysis using the emmeans package in R. We report the marginal mean as estimate and 95 % Confidence Interval.

Emmeans Analysis of linear models on NBES						
**Factor**	**Level**	**Estimate**	**SE**	**df**	**Lower CI**	**Upper CI**
Richness	2	1.830	0.175	135	1.484	2.176
	4	-0.126	0.248	135	-0.616	0.363
	5	0.418	0.554	135	-0.677	1.513
Temp	Fluc	0.7898	0.364	135	0.0691	1.510
	Inc	0.0685	0.364	135	-0.6521	0.789
	Inc+Fluc	1.2632	0.364	135	0.5425	1.984

SupplementaryTable S4. Overview of parameter values and variables used in the model simulations.

**Variable or parameter**	**Description**	**Value/ Formula**
**Species level variables and parameters**		
$\boldsymbol{N_i(t0)}$	The biomass of species *i* at time *t0*	0.1; same for all species in all simulated communities.
$\boldsymbol{b_{opt,i}}$	Temperature optimum of birth rate of species *i*	Either all species had the same $\boldsymbol{b_{opt,i}}$ = 17.5°C or all species had different $\boldsymbol{b_{opt,i}}$ that were evenly distributed between 15°C and 20°C.
$\boldsymbol{a_{b,i}}$	Birth rate at optimum temperature	1; same for all species in all simulated communities.
$\boldsymbol{s_i}$	Width of temperature response curve of birth rate.	30; same for all species in all simulated communities.
$\boldsymbol{a_{d,i}}$	Constant of temperature-death rate function	0.01; same for all species in all simulated communities.
$\boldsymbol{z}$	Slope of temperature-death rate function.	0.2; same for all species in all simulated communities.
$\boldsymbol{\beta, \delta}$	Density dependent constants for species carrying capacity.	0.025; same for all species in all simulated communities.
**Community level parameters**		

α_{ij}	The strength of interspecific interaction between species *i* and *j*.	Absolute value of draws from a normal distribution with mean 0 and standard deviation α_{ij}_sd
α_{ij}_sd	Strength of interspecific competition in a community.	Sequence of 0, 0.25, 0.5. Low values create a community with weak competitive interactions, high values create a community with strong interactions.
S	Species richness	5, same for all communities
T	Temperature	Control = 17.5°C<br>Increase = 15 (Temperature minimum) and 20°C (Temperature maximum)<br>Fluctuations = 17.5°C (Mean temperature) $\pm$ 2.5 °C

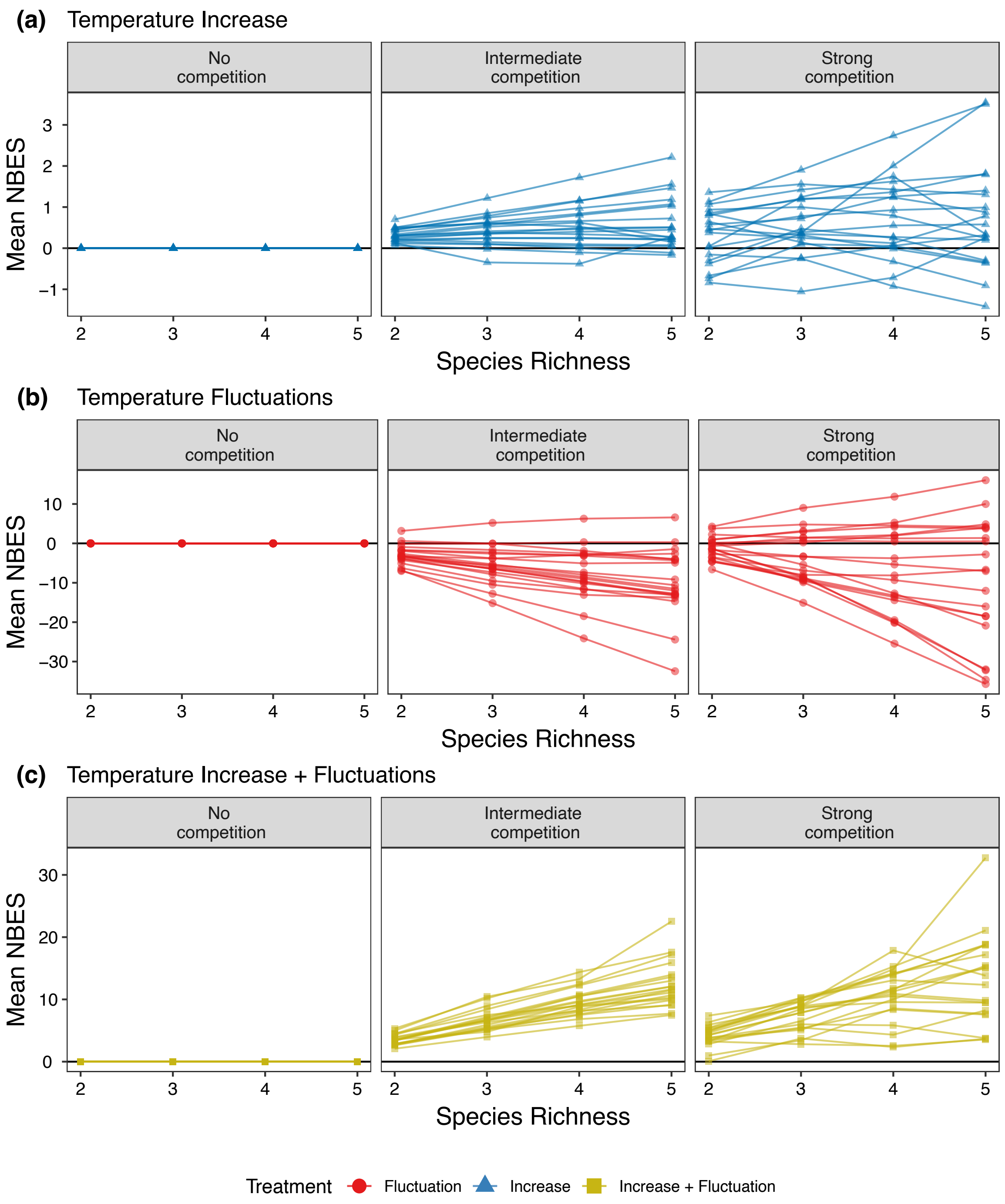

Supplementary Fig. S1: Results of the model simulations for communities where species had different temperature optima (TOpt ranging between 15-20 °C). Mean net biodiversity effect on stability (NBES) for each model run and species richness level under (a) increasing temperatures (press), (b) temperature fluctuations, and (c) the combination of temperature fluctuations and temperature increase. We calculated mean NBES across species combinations for each model run separately (n=10 for richness levels 2 and 3, n = 5 for richness level 4, and n =1 for richness level 5). The competition strength determined the magnitude of the NBES, while the direction of the NBES was dependent on the temperature optima distribution. Different colors and shapes indicate different disturbance regimes, different facets indicate different competition strength where higher values indicate stronger interspecific competition.

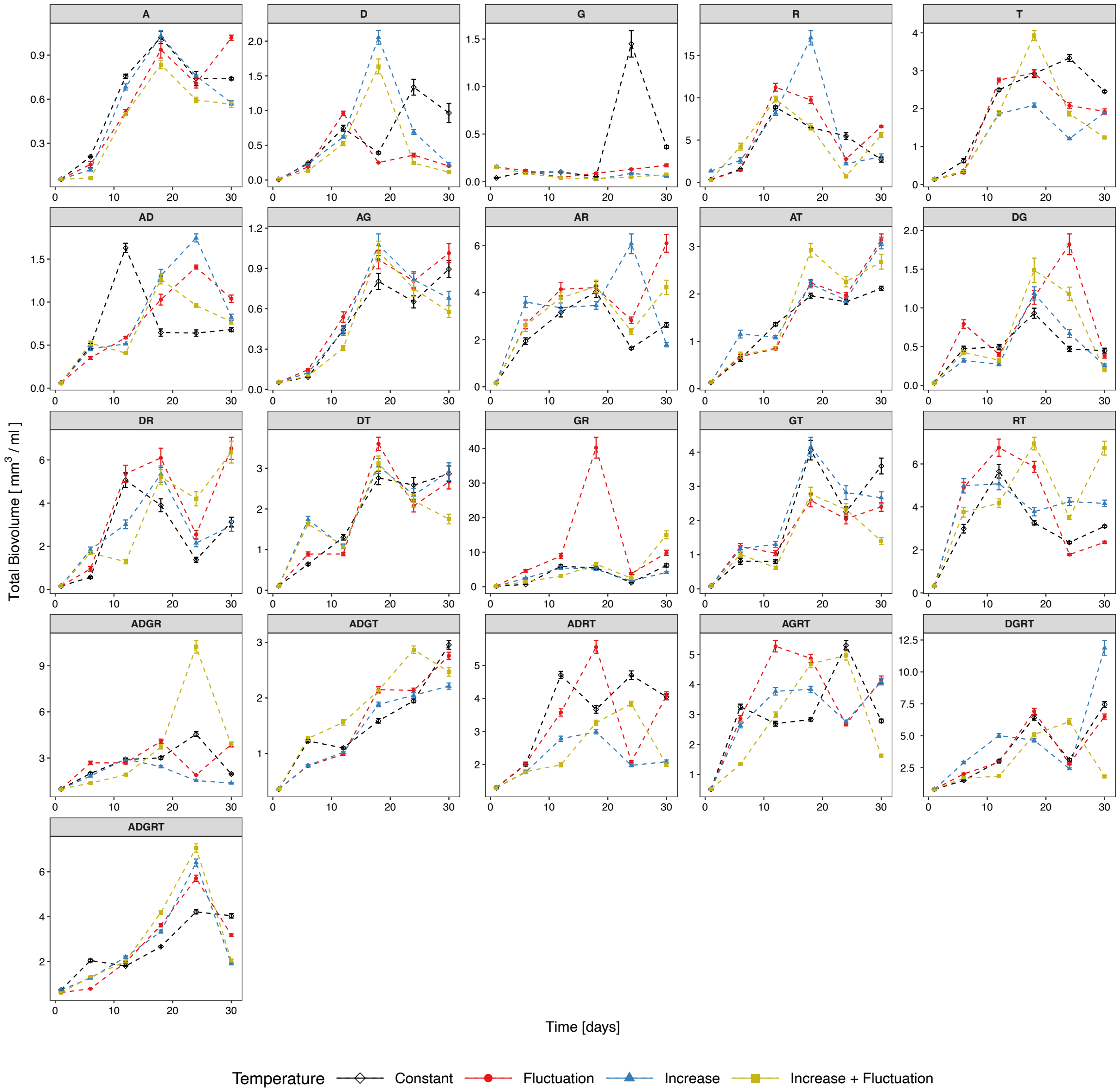

Supplementary Fig. S2. Total biomass of species assemblages over time. Mean (± SE, *n* = 3) total biomass was measured from the total biovolume of the respective species compositions over time. Depending on species composition, temperature treatments frequently enhanced biomass production over time, except for most monocultures where temperature treatments decreased biomass compared to the constant temperature control. Species are abbreviated as A – *Asterionellopsis*, D – *Ditylum*, G – *Guinardia*, R – *Rhizosolenia*, T – *Thalassionema*, and species combinations are indicated by a combination of these abbreviations in each panel. Richness levels comprised species in monoculture (top row), two species (second and third row), four species (fourth row), and five species (bottom row).

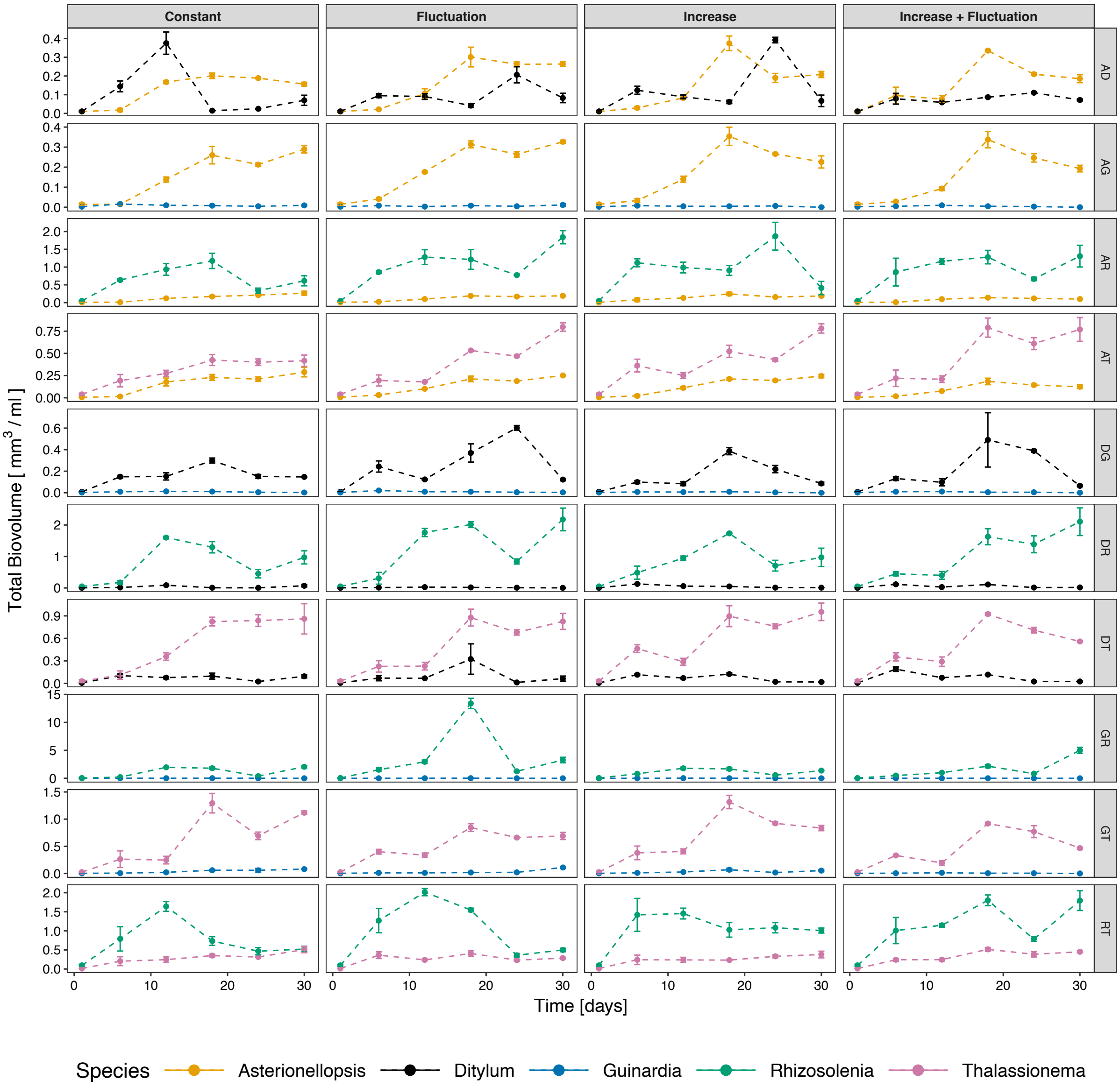

Supplementary Fig. S3. Species-specific biomass of two species in mixtures over time. Mean (± SE, n = 3) total biovolume of each species in the two species combinations. Species in combinations are abbreviated as A – *Asterionellopsis*, D – *Ditylum*, G – *Guinardia*, R – *Rhizosolenia*, T – *Thalassionema*.

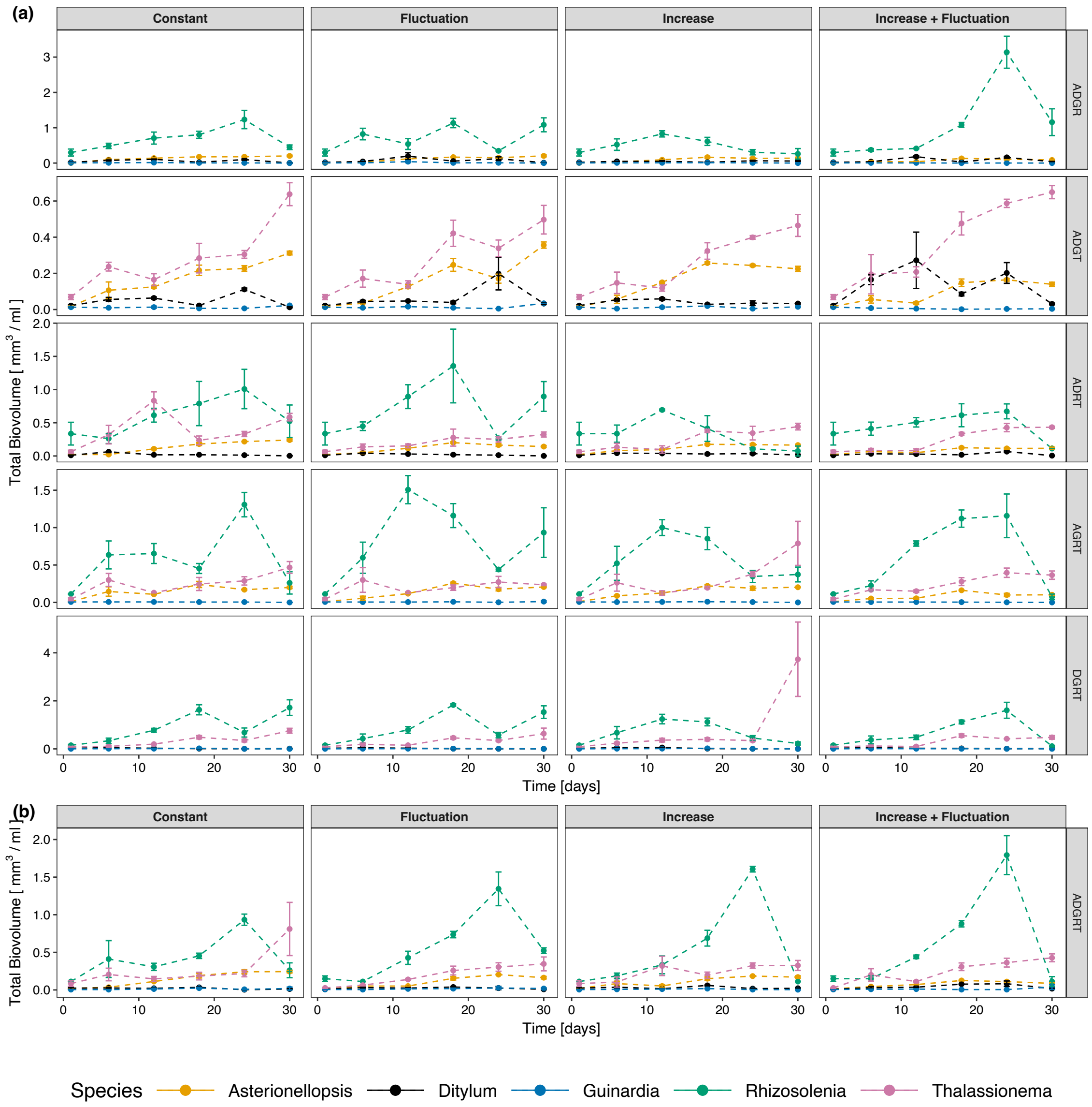

Supplementary Fig. S4. Species-specific biomass in four-species and five-species assemblages over time. Mean (± SE, n = 3) total biovolume of each species in the four species (a) and five species assemblages (b). Species in combinations are abbreviated as A – *Asterionellopsis*, D – *Ditylum*, G – *Guinardia*, R – *Rhizosolenia*, T – *Thalassionema*.

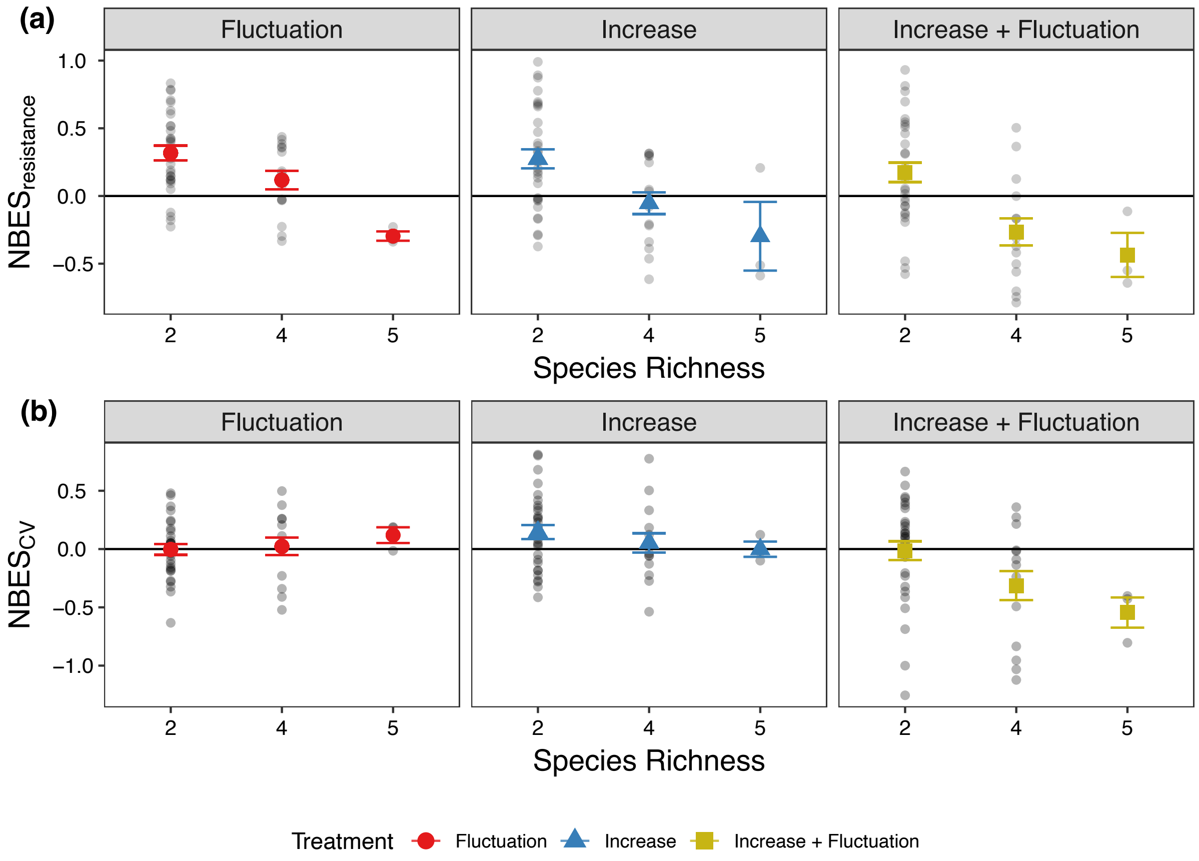

Supplementary Fig. S5. The Net Biodiversity Effect on Stability for temporal variability and resistance. Mean (n = 3 ± SE) net biodiversity effect on stability (NBES) based on resistance (a) and temporal variability (b) in our phytoplankton microcosms. Resistance was calculated as the difference in response ratios of multi-species assemblages compared to what is expected (a). Temporal variability was calculated as the difference in the coefficient of variation (CV) of the absolute response ratios of multi-species assemblages compared to what is expected from monoculture, over the species richness gradient (b). Raw values are given as black dots. Different facets indicate different disturbance treatments, also highlighted by colours and shapes.

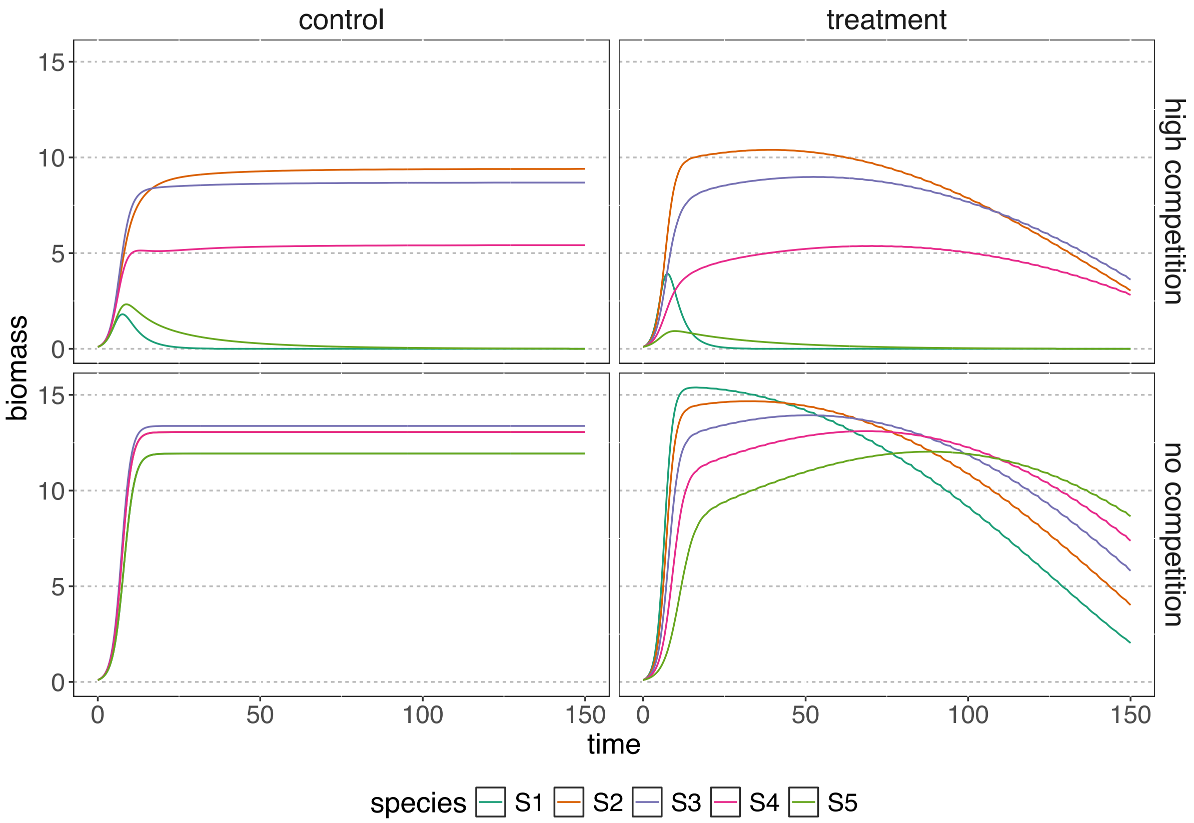

Supplementary Fig. S6: Exemplary model runs of two communities where all species have different thermal optima and either strong competition or no competition under increasing temperatures and control conditions.

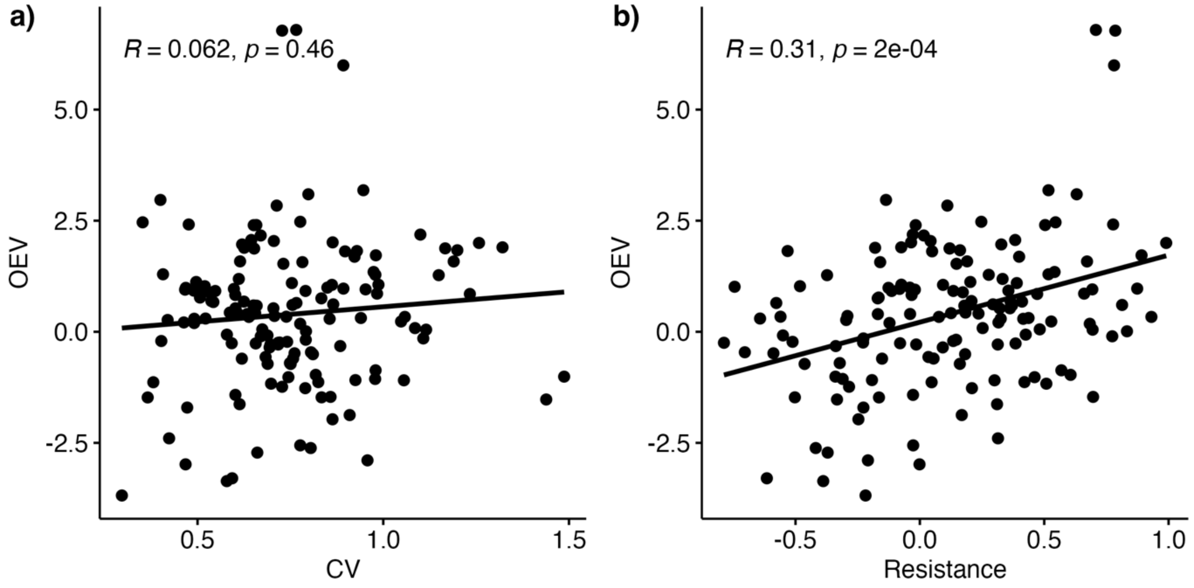

Supplementary Fig. S7: Spearman rank correlation between OEV and temporal variability. Temporal variability was measured as the Coefficent of Variation (CV) of the response ratio over time (a). Resistance was measured as the response ratio after initiation of the disturbance at t1 (see Methods section for calculation details) (b). Correlation results highlight the multidimensional nature of the OEV index. One point represents one replicate (n=3) of an experimental community with S = 2, S=4, S=5 species.
