# Supplementary material for: Quantifying the net effect of biodiversity on ecological stability
Source: Nat Commun. 2026 Jul 7;17:5942. doi: 10.1038/s41467-026-75047-z (PMC13341745; doi:10.1038/s41467-026-75047-z)
Supplement: Supplementary file 2 — Reporting Summary [file 41467_2026_75047_MOESM2_ESM.pdf]

nature portfolio

Corresponding author(s): Charlotte Kunze

Last updated by author(s): Nov 19, 2025

Reporting Summary

Nature Portfolio wishes to improve the reproducibility of the work that we publish. This form provides structure for consistency and transparency in reporting. For further information on Nature Portfolio policies, see our Editorial Policies and the Editorial Policy Checklist.

Statistics

For all statistical analyses, confirm that the following items are present in the figure legend, table legend, main text, or Methods section.

n/a	Confirmed	
☐	☒	The exact sample size (*n*) for each experimental group/condition, given as a discrete number and unit of measurement
☐	☒	A statement on whether measurements were taken from distinct samples or whether the same sample was measured repeatedly
☐	☒	The statistical test(s) used AND whether they are one- or two-sided<br>*Only common tests should be described solely by name; describe more complex techniques in the Methods section.*
☐	☒	A description of all covariates tested
☒	☐	A description of any assumptions or corrections, such as tests of normality and adjustment for multiple comparisons
☐	☒	A full description of the statistical parameters including central tendency (e.g. means) or other basic estimates (e.g. regression coefficient) AND variation (e.g. standard deviation) or associated estimates of uncertainty (e.g. confidence intervals)
☐	☒	For null hypothesis testing, the test statistic (e.g. *F*, *t*, *r*) with confidence intervals, effect sizes, degrees of freedom and *P* value noted<br>*Give P values as exact values whenever suitable.*
☒	☐	For Bayesian analysis, information on the choice of priors and Markov chain Monte Carlo settings
☒	☐	For hierarchical and complex designs, identification of the appropriate level for tests and full reporting of outcomes
☒	☐	Estimates of effect sizes (e.g. Cohen's *d*, Pearson's *r*), indicating how they were calculated

Our web collection on statistics for biologists contains articles on many of the points above.

Software and code

Policy information about availability of computer code

Data collection	Conceptual figures were created using Inkscape 1.2 (version dc2aeda, 2022-05-15). Model Simulations were done in R version 4.4.3 using packages ggbeeswarm (0.7.3), foreach (1.5.2), svMisc (1.4.3), parallel, here(1.0.2) and tidyverse (2.0.0). Simulated data are permanently archived on Zenodo https://doi.org/10.5281/zenodo.15274625.
Data analysis	All analyses were conducted using the R programming language in R version 4.5.3 using the additional packages MESS (0.6.0), cowplot (1.2.0), patchwork (1.3.2), ggpubr (0.6.3). All code used for the analysis is permanently archived on Zenodo. The analysis of experimental data can be accessed under https://doi.org/10.5281/zenodo.19495106 and the simulation analysis under https://doi.org/10.5281/zenodo.19495091

For manuscripts utilizing custom algorithms or software that are central to the research but not yet described in published literature, software must be made available to editors and reviewers. We strongly encourage code deposition in a community repository (e.g. GitHub). See the Nature Portfolio guidelines for submitting code & software for further information.

Data

Policy information about availability of data

All manuscripts must include a data availability statement. This statement should provide the following information, where applicable:

- Accession codes, unique identifiers, or web links for publicly available datasets
- A description of any restrictions on data availability
- For clinical datasets or third party data, please ensure that the statement adheres to our policy

Data of the experiment, including all raw data are archived on Figshare: https://doi.org/10.6084/m9.figshare.25568490.v3. Simulated data are archived on Zenodo: https://doi.org/10.5281/zenodo.15274625

Research involving human participants, their data, or biological material

Policy information about studies with human participants or human data. See also policy information about sex, gender (identity/presentation), and sexual orientation and race, ethnicity and racism.

Reporting on sex and gender	*Use the terms sex (biological attribute) and gender (shaped by social and cultural circumstances) carefully in order to avoid confusing both terms. Indicate if findings apply to only one sex or gender; describe whether sex and gender were considered in study design; whether sex and/or gender was determined based on self-reporting or assigned and methods used. Provide in the source data disaggregated sex and gender data, where this information has been collected, and if consent has been obtained for sharing of individual-level data; provide overall numbers in this Reporting Summary. Please state if this information has not been collected. Report sex- and gender-based analyses where performed, justify reasons for lack of sex- and gender-based analysis.*
Reporting on race, ethnicity, or other socially relevant groupings	*Please specify the socially constructed or socially relevant categorization variable(s) used in your manuscript and explain why they were used. Please note that such variables should not be used as proxies for other socially constructed/relevant variables (for example, race or ethnicity should not be used as a proxy for socioeconomic status). Provide clear definitions of the relevant terms used, how they were provided (by the participants/respondents, the researchers, or third parties), and the method(s) used to classify people into the different categories (e.g. self-report, census or administrative data, social media data, etc.) Please provide details about how you controlled for confounding variables in your analyses.*
Population characteristics	*Describe the covariate-relevant population characteristics of the human research participants (e.g. age, genotypic information, past and current diagnosis and treatment categories). If you filled out the behavioural & social sciences study design questions and have nothing to add here, write "See above."*
Recruitment	*Describe how participants were recruited. Outline any potential self-selection bias or other biases that may be present and how these are likely to impact results.*
Ethics oversight	*Identify the organization(s) that approved the study protocol.*

Note that full information on the approval of the study protocol must also be provided in the manuscript.

Field-specific reporting

Please select the one below that is the best fit for your research. If you are not sure, read the appropriate sections before making your selection.

☐ Life sciences ☐ Behavioural & social sciences ☒ Ecological, evolutionary & environmental sciences

For a reference copy of the document with all sections, see nature.com/documents/nr-reporting-summary-flat.pdf

Ecological, evolutionary & environmental sciences study design

All studies must disclose on these points even when the disclosure is negative.

Study description	For the experimental part of the study, we manipulated phytoplankton species richness and temperature treatment in a full-factorial design. We used four levels of species richness, that is monocultures of all species, two-species, four-species, and five-species communities for all possible combinations of species,nesting species combinations in species richness levels. All possible combinations were exposed to four different temperature treatments, i.e. diurnal temperature fluctuations, temperature increase, constant temperatures and diurnal fluctuations around an increasing mean. Each combination was replicated three times.
Research sample	Five phytoplankton species extracted from the North sea that is Asterionellopsis glacialis, Ditylum brightwelii, Guinardia striata, Thalassionema nitzschioides, and Rhizosolenia setigera. The marine diatoms were isolated in 2017 and kept in the lab at 18 degree prior experimental start. Experimental communities were assembled in a substitution design using 50 ml cell culture flasks. We chose these species because of their large variation in cell size, which is known to be a master trait in phytoplankton, influencing differences in growth rates, responses to temperature, and nutrient prevalence. In addition, diatoms play a key role in marine primary production worldwide. We sampled every 6 days on days 0, 6, 12, 18, 24, 30 to capture community dynamics following

temperature treatments.

Sampling strategy	We employed a fully factorial design, replicating each species combination and temperature treatment three times. This replication level was chosen to balance experimental feasibility with the need for sufficient statistical power, ensuring that all samples could be processed within a single day while capturing variability across treatments. We decided to replicate each species combination and temperature treatment three times to ensure that the experiment was feasible to conduct and all samples could be processed within one day.
Data collection	Charlotte Kunze sampled all experimental units and fixated the samples in lugols iodine solution. For sampling, experimental units were taken out of the water bath, stirred gently to homogenise the culture and a sample of 0.5 ml transferred into 48-well plates (Sarstedt). Toni Schott counted all experimental samples within one month following the end of the experiment using an inverted microscope and noting the counts on printed paper tables with a pen.
Timing and spatial scale	The experiment started on 19 January 2022 and lasted 30 days ending on 18 February 2022. We sampled every 6 days from the 50 ml microcosms on days 0, 6, 12, 18, 24, 30 to capture community dynamics following temperature treatments.
Data exclusions	No data were excluded from the analysis.
Reproducibility	For reproducibility, we documented the experimental set-up in detail, monitored temperature for temperature treatments carefully, and documented measurement procedures. Temperature treatments were created using water baths which were heated using pre-programmed temperature curves. Phytoplankton organisms were chosen to be very abundant in the North sea. Independent replicates were conducted under the same conditions, and key findings were cross-checked for consistency. Data analysis scripts and raw data were archived for transparency and re-analysis.
Randomization	Culture flasks were randomly assigned to one of the four treatments, and their arrangement within the mesocosms was randomized. Mesocosms themselves were also randomly allocated to the respective treatments to minimize spatial and experimental biases.
Blinding	Blinding was not applied during data acquisition or analysis because treatment groups were visibly distinguishable, making blinding impractical. However, standardized protocols were followed to ensure objectivity and consistency in measurements.

Did the study involve field work? ☐ Yes ☒ No

Reporting for specific materials, systems and methods

We require information from authors about some types of materials, experimental systems and methods used in many studies. Here, indicate whether each material, system or method listed is relevant to your study. If you are not sure if a list item applies to your research, read the appropriate section before selecting a response.

Materials & experimental systems

n/a	Involved in the study	
☒	☐	Antibodies
☒	☐	Eukaryotic cell lines
☒	☐	Palaeontology and archaeology
☒	☐	Animals and other organisms
☒	☐	Clinical data
☒	☐	Dual use research of concern
☐	☒	Plants

Methods

n/a	Involved in the study	
☒	☐	ChIP-seq
☒	☐	Flow cytometry
☒	☐	MRI-based neuroimaging

Dual use research of concern

Policy information about dual use research of concern

Hazards

Could the accidental, deliberate or reckless misuse of agents or technologies generated in the work, or the application of information presented in the manuscript, pose a threat to:

No	Yes	
☒	☐	Public health
☒	☐	National security
☒	☐	Crops and/or livestock
☒	☐	Ecosystems
☒	☐	Any other significant area

Experiments of concern

Does the work involve any of these experiments of concern:

No	Yes	
☒	☐	Demonstrate how to render a vaccine ineffective
☒	☐	Confer resistance to therapeutically useful antibiotics or antiviral agents
☒	☐	Enhance the virulence of a pathogen or render a nonpathogen virulent
☒	☐	Increase transmissibility of a pathogen
☒	☐	Alter the host range of a pathogen
☒	☐	Enable evasion of diagnostic/detection modalities
☒	☐	Enable the weaponization of a biological agent or toxin
☒	☐	Any other potentially harmful combination of experiments and agents

Plants

Seed stocks	We used five different phytoplankton species isolated from the North Sea in summer 2017 that were maintained at 18 degree constant in our culture collection at 25 PSU artificial sea water: Asterionellopsis glacialis, Ditylum brightwelii , Guinardia striata, Thalassionema nitzschioides, and Rhizosolenia setigera.
Novel plant genotypes	n/a
Authentication	n/a
