# Supplementary material for: Quantifying the net effect of biodiversity on ecological stability
Source: Nat Commun. 2026 Jul 7;17:5942. doi: 10.1038/s41467-026-75047-z (PMC13341745; doi:10.1038/s41467-026-75047-z)
Supplement: Supplementary file 3 — Transparent Peer Review file [file 41467_2026_75047_MOESM3_ESM.pdf]

nature portfolio

Peer Review File

Quantifying the net effect of biodiversity on ecological stability

Corresponding Author: Dr Charlotte Kunze

This file contains all reviewer reports in order by version, followed by all author rebuttals in order by version.

Version 0:

Reviewer comments:

Reviewer #1

(Remarks to the Author)
The goal of the present study is to quantify net biodiversity effects on various dimensions of ecological stability (NBES). While net biodiversity effects on functioning have been instrumental for biodiversity-ecosystem functioning research, an analogous metric for ecological stability has so far been missing. Therefore, this study addresses an important and timely issue that is of broad significance for biodiversity-stability research.

The proposed definition of NBES is based on the difference between observed and expected stability comparing standardized response ratios between treatment and control. This results in negative/positive NBES if the difference between observed and expected is negative/positive. The expected stability is derived from the observed monoculture stability, which is scaled to mixture richness considering the initial proportions of all species in a community.
The NBES metric can be calculated for a range of stability indicators, such temporal stability, resistance or recovery. In the study, it is applied to the overall ecological vulnerability index (OEV), which is based on biomass change through time and quantified by the area under the response ratio curve.

The approach is subsequently tested with a simulation study to understand the effects of temperature regime and richness on community biomass and resulting NBES and compared to an experiment that manipulates species richness and composition of phytoplankton communities that are exposed to 4 different temperature regimes: constant temperature control, temperature fluctuations, a trend (increasing temperature), and a trend coupled with diurnal temperature fluctuations.

While I see the merits of the proposed NBES approach, it is pitched to be akin to net biodiversity effects on functioning. However, the approach does not allow for partitioning stability into complementarity and selection effects, nor mechanisms underlying particular dimensions of stability, such as asynchrony or the effects of species interactions on average population biomass in the case of temporal stability. This is partly due to the flexibility regarding the chosen stability metric, which may be underpinned by different mechanisms. Subsequent statistical analysis may allow inference on mechanisms, however, without the conceptual clarity of the additive partitioning by Loreau & Hector and its ability to pinpoint mechanisms. Despite these downsides, the merits outweigh the limitations, providing biodiversity scientists with a tool to synthesise biodiversity effects across a plethora of stability metrics.

Below I have made a range of comments to clarify the calculation of the metric and the interpretation of the simulation and experimental results, which I sometimes found counterintuitive.

Metric:
According to Fig 1, for the mixture expectation, you compare the expected treatment stability to the observed control stability, as for the two hypothetical observed mixtures. However, would it not also make sense to calculate the expected stability in the control and compare it to the expected stability in the treatment to generate the expected stability? Have you explored using this expected versus observed control stability and how this affects your interpretation of NBES?

Simulations:
I am a bit puzzled by the result that positive NBES manifest consistently when there is no variation in thermal optima, while there are positive and negative effects with variation in optima. Maybe this is due to all optima being at 17.5 C which is in the mid-range of the temperature trend (and the other regimes not shown in Fig 2)? I would expect negative NBES if you would

vary the optima to say 15 C, because then the lack of response diversity would be detrimental when temperatures increase or fluctuate around a higher mean.

Experiments:
lines 213-214: I suggest an analysis that would test the richness effect (either continuous or as a factor), the temperature regime and their interaction, with composition as a random effect. This seems more aligned with the emphasis on richness in the simulation models. The imbalanced number of compositions should not be an issue for a mixed model.

Other comments:

Line 36: "extend" suggests that you used a partition that allows split stability contributions into complementarity and selection effects, which is not the case. Suggest to state: "Here we develop an approach to quantify NBES, inspired by / drawing on Loreau & Hectors's approach to assess ..."

lines 55-61: I understand that these hypotheses apply to both simulations and experiments; however, at the start of the discussion you check whether they apply to either the experiment or the simulation, judging that there is some support when either is matched. Please evaluate your hypotheses for both simultaneously (only supported when both are met), matching paired hypotheses outlined in lines 55-61.

line 104: clarify/state how to standardise the response ratio

line 136: use trend or increasing temperature instead of "pressure"

line 139: delete "significant" since this seems to be derived from visual rather than statistical inspection (also not recommending the latter!)

line 140-141: "differed among disturbance types" seem to contradict the caption of Fig 2 where you state "only results from the increasing temperature are shown this disturbances regimes were similar"; did you mean variation in thermal optima?

lines 142-150 are rather descriptive and do not provide explanations for the observed patterns. Suggest to add some explanation for these patterns here or in the discussion.

line 164: suggest to use only optimum/optima rather than "temperature preference(s)" for consistency here and throughout the ms

line 306: do you mean Fig S5?

line 382: Food webs are not an individual system (unless you refer to one specifically). Maybe refer to grasslands rather than food webs?

line 445: did you discard observations to disregard the effect of transients in the simulations?

Figures:

Fig 2:
Better to state alpha SD as the facet labels; only after reading the methods I realized that alpha refers to the SD and not the mean.
Fix "temperature" in panel title b)
Suggest to alert readers to the different Y axes scales: the positive NBES in panel a are consistent but much weaker than the NBES in panel b

Fig 3:
I have a hard time telling apart the 4 and 5 species communities in the experiments. Maybe use a different colour gradient or a different symbol for richness level 5?

Fig 4:
Caption: Not clear what is meant by "indicate greater stability with positive and negative deviations?" Suggest only explaining what high and low observed OEV means in terms of stability.

(Remarks on code availability)

Reviewer #2

(Remarks to the Author)
Key results and significance

This paper addresses the question of quantifying the effects of biodiversity on stability. This is a relevant and timely topic,

and it is still understudied; therefore this study aims at filling an important gap. This goal is pursued by extending the framework developed by Loreau & Hector (ref. 19 in the manuscript) to asses the effect of diversity on ecosystem functioning. The authors define an index, the net biodiversity effect on functional stability (NBES), which quantifies the difference between observed and expected stability and can be computed starting from different metric of stability measurable by comparing a treatment response to a control. The NBES is then measured in simulations of a temperature-dependent Lotka-Volterra competition model and in microcosm experiment involving 5 diatom species grown in isolation and in all the possible combinations creating communities of 2, 4, and 5 species. The author reports that:

1. Simulated communities showed sometimes lower and sometimes greater stability than expected. Experimental communities showed overall greater stability than expected.

2. The NBES varied in direction and magnitude in the simulations. In the experiments, NBES was highest for two-species combinations and decreased at higher species richness levels.

3. The NBES varied among species combinations and temperature treatments according to species thermal optima in both simulations and the experiment, and even became negative for some.

4. In simulations, the magnitude of the NBES varied with competition strength.

The problem addressed by the authors is relevant and the approach is clear and potentially productive. I have, however, some major and minor comments that I detail below.

Major comments

- The OVE is the main (in)stability proxy used in the paper, it has been devised to combine multiple dimensions of post-disturbance stability into a single measure of instability. Why this is the case, however, is not clearly described in the context of this work. While its operational definition is clear, its connections with different measure of instability is lacking. There are several references to reference 20, where it was introduced in 2021, but to the best of my understanding, it is not (yet) such a broadly used and known index that can afford to be carefully described in a work such as the present one. I think it is important to make a case also in the present work about how OVE explicitly connects to different dimensions of (in)stability and why it summarizes them meaningfully.

- The NBES results for the simulations in the case alpha=0 are potentially problematic, or, at best, hard to interpret. Following the model definition, the case alpha=0 correspond to an ensamble of non-interacting species. Therefore, by definition, biodiversity should not matter and should not show any net effect on stability. However, Figure 2 b and Figure 3 a and c show such effects, raising doubts on the reliability of the method and the interpretation of the NBES index.

- I have some reservation on part of the interpretation of the data results on OVE and on NBES. Focusing on Figure 4 and on lines 193 to 197, while it is true that there is variability in the observed OVE, it does not seem the case that "the majority of multi species combinations showed positive or near-neutral deviations from the constant temperature controls". It seems to me that, while there is variability there are overall neutral deviations from the constant temperature control in the multispecies communities, with the exception of the case of 2 species where indeed there seems to be an overall positive trend. Regarding NBES (Figure 5), panel b for 4 species shows a dependence of the effects on the specific combinations rather than the overall number of species. For example the claim that the combined condition correspond to an overall posititve effect is biased by the high positive value of the combination ADGT, but it does not reflect the fact that actually for 3 combination out of 5 there is a negative effect.

- Based on the results from the experiments, I do not fully agree with the conclusions regarding the 4 hypothesis that this paper addresses. H1 could be considered partly accepted, I agree, however, as pointed out in the previous comment the overall positive effect seen in Figure 5 a could be due to specific combination of species, rather than by the biodiversity itself. Regarding H2, "NBES increases with increasing species richness" I find a bit harder to consider it partly accepted, apart from the results that communities with two species seems to have a positive NBES, the observations seems to point towards the opposite, with this positive effect vanishing when the number of species increases. I am not fully convinced about H3. from Figure 3 b, d and e is not clear that the variation observed is due to thermal optima or other confunders. I am not necessarily suggesting that this is not the case, however there is space to improve on this point and clarify the arguments that lead to this conclusion in the result section. H4 is only addressed by simulation and therefore it should have a different (maybe lower) status with respect to the other hypothesis in my opinion.

Minor comments

- I believe the paper would benefit in clarity and impact if the experimental section is given more relevance by being discussed first in the results, with the simulations discussed only after it.

- It should be stated explicitly somewhere that the there are 1s on the diagonal of the interaction matrix of the Lotka-Volterra model.

Considerations on data and code availability, references to previous literature

Data and code are available and relevant previous literature is appropriately acknowledged. I do not have the expertise to

judge the appropriateness and robustness of the experimental procedures.

(Remarks on code availability)

Version 1:

Reviewer comments:

Reviewer #1

(Remarks to the Author)
Overall, my concerns have been thoroughly addressed, and I feel that after this round of revision, the manuscript has improved both in clarity and in providing a more nuanced picture of the findings. This work provides an important advance for empiricists and theoreticians to understand the multifaceted relationships between species richness and traits mediating the stability of communities to climate change.

When re-reading the manuscript, I noted a few minor (editorial) issues that should be fixed before moving forward.

Minor issues:
- Replace pressure with disturbance throughout the ms
- lines 483-484: explain what you mean by "favourable optimum"? Small variation around a mean that is close to the expected average temperature change?
- Figure 2 panel b title: there is still a typo (Tempterature)
- Figure 3: state more explicitly that the Y axis is the average bopt across communities, since the caption states that species had variable bopt
- line 559: the figure suggests lower temporal stability; suggest deleting the statement in the parenthesis, which may be misinterpreted as higher temporal stability in the more species-rich communities.
- line 566: replace "gradual warming" with "temperature gradient of constant temperatures".
- line 589-593: This could also be interpreted oppositely: if you know the thermal optima of your community, you may be able to predict the community response given a know thermal regime and interaction structure. The composition effect is only idiosyncratic if you do not know the mapping between species identity and thermal optimum and the interaction structure, suggesting that predicting community responses to climate change crucially hinges on getting better estimates of these components across natural communities.

(Remarks on code availability)

Reviewer #2

(Remarks to the Author)
I appreciate the effort that the authors put into addressing my comments. And I am satisfied with all the reviews, except for one point.

I still believe that hypothesis 4 (H4) should notbe included with the others that have been explored experimentally, as it remains analyzed only through simulations. I expressed this concern, but it was not addressed.

I am not suggesting discarding the discussion on that point entirely. I think that the main hypothesis addressed formally should be the first 3, and a discussion about that aspect can find its place in the text while discussing the outcomes of the model, but not on the same level as the other hypotheses (in the intro and in the conclusions).

I think that rather than weakening the impact of the paper, clarifying this point will bring more clarity to what is being achieved and do a better service to the broader community.

(Remarks on code availability)

Open Access This Peer Review File is licensed under a Creative Commons Attribution 4.0 International License, which permits use, sharing, adaptation, distribution and reproduction in any medium or format, as long as you give appropriate credit to the original author(s) and the source, provide a link to the Creative Commons license, and indicate if changes were made.
In cases where reviewers are anonymous, credit should be given to 'Anonymous Referee' and the source.
The images or other third party material in this Peer Review File are included in the article's Creative Commons license, unless indicated otherwise in a credit line to the material. If material is not included in the article's Creative Commons license and your intended use is not permitted by statutory regulation or exceeds the permitted use, you will need to obtain permission directly from the copyright holder.
To view a copy of this license, visit https://creativecommons.org/licenses/by/4.0/

Response letter

REVIEWER COMMENTS

Reviewer #1 (Remarks to the Author):

The goal of the present study is to quantify net biodiversity effects on various dimensions of ecological stability (NBES). While net biodiversity effects on functioning have been instrumental for biodiversity-ecosystem functioning research, an analogous metric for ecological stability has so far been missing. Therefore, this study addresses an important and timely issue that is of broad significance for biodiversity-stability research.

The proposed definition of NBES is based on the difference between observed and expected stability comparing standardized response ratios between treatment and control. This results in negative/positive NBES if the difference between observed and expected is negative/positive. The expected stability is derived from the observed monoculture stability, which is scaled to mixture richness considering the initial proportions of all species in a community.
The NBES metric can be calculated for a range of stability indicators, such temporal stability, resistance or recovery. In the study, it is applied to the overall ecological vulnerability index (OEV), which is based on biomass change through time and quantified by the area under the response ratio curve.

The approach is subsequently tested with a simulation study to understand the effects of temperature regime and richness on community biomass and resulting NBES and compared to an experiment that manipulates species richness and composition of phytoplankton communities that are exposed to 4 different temperature regimes: constant temperature control, temperature fluctuations, a trend (increasing temperature), and a trend coupled with diurnal temperature fluctuations.

While I see the merits of the proposed NBES approach, it is pitched to be akin to net biodiversity effects on functioning. However, the approach does not allow for partitioning stability into complementarity and selection effects, nor mechanisms underlying particular dimensions of stability, such as asynchrony or the effects of species interactions on average population biomass in the case of temporal stability. This is partly due to the flexibility regarding the chosen stability metric, which may be underpinned by different mechanisms. Subsequent statistical analysis may allow inference on mechanisms, however, without the conceptual clarity of the additive partitioning by Loreau & Hector and its ability to pinpoint mechanisms. Despite these downsides, the merits outweigh the limitations, providing biodiversity scientists with a tool to synthesise biodiversity effects across a plethora of stability metrics.

Below I have made a range of comments to clarify the calculation of the metric and the interpretation of the simulation and experimental results, which I sometimes found counterintuitive.

We are glad to hear that the reviewer found the approach useful and are thankful for the constructive comments. We are confident that the changes that we have made address the comments and concerns raised by the reviewer. We aimed to improve the clarity of the current approach while better introducing its advantages. We are aware that the calculation in relative space has its downsides but are confident that the comparison to an undisturbed control nonetheless provides a flexible framework to disentangle disturbance effects on diversity-stability relationships.

Metric:
According to Fig 1, for the mixture expectation, you compare the expected treatment stability to the observed control stability, as for the two hypothetical observed mixtures. **However, would it not also make sense to calculate the expected stability in the control and compare it to the expected stability in the treatment to generate the expected stability? Have you explored using this expected versus observed control stability and how this affects your interpretation of NBES?**
In our approach we use a comparison of a disturbed community to an undisturbed community as the foundation of our stability calculation. This allows accounting for variations in biomass that are not a result of the introduced treatments (but over time) and represents a comparison of the difference between disturbance and control. Since the (in-)stability is already the difference between treatment and control, we cannot compare it to fluctuations in the control itself. We have added a paragraph to the Methods section (LL619-623): "Comparing the dynamics of a disturbed community to those of an undisturbed control allows us to account for natural temporal variation in biomass that is unrelated to the applied disturbance and isolates the difference attributable to the treatment itself. Because instability is defined as the difference between disturbance and control trajectories, it is not meaningful to further compare this quantity to fluctuations occurring within the control community alone." As well as Box1 (LL138-141): "By comparing the dynamics of a disturbed community with those of an undisturbed control community, it is possible to account for natural variations in biomass that are unrelated to the applied disturbance, and thereby to isolate the difference attributable to the treatment itself."

Simulations:
I am a bit puzzled by the result that positive NBES manifest consistently when there is no variation in thermal optima, while there are positive and negative effects with variation in optima. Maybe this is due to all optima being at 17.5 C which is in the mid-range of the temperature trend (and the other regimes not shown in Fig 2)? I would expect negative NBES if you would vary the optima to say 15 C, because then the lack of response diversity would be detrimental when temperatures increase or fluctuate around a higher mean.
Yes, that's exactly the case. We chose to use a thermal optimum at 17.5 for the scenario "no diversity in thermal optima" (all species have same Topt) to allow a comparison with the scenario where there is "high diversity in temperature optima" (each species has different Topt). In the latter, the mean temperature optimum (across all species in a community) is 17.5 degree. If we were to set the temperature optima at 15 degree in the "no diversity in temperature optima" scenario instead, we would observe negative NBES. In response to this comment, we have changed the sentence in the results as follows (LL185-187): "When all species had the same temperature optimum of 17.5 °C – near the regime temperature - the NBES was consistently positive and increased with increasing species richness (Fig. 2)"

Experiments:
lines 213-214: I suggest an analysis that would test the richness effect (either continuous or as a factor), the temperature regime and their interaction, with composition as a random effect. This seems more aligned with the emphasis on richness in the simulation models. The imbalanced number of compositions should not be an issue for a mixed model.
We agree that this is a useful addition for the discussion of our results and have conducted such an analysis with temperature regime and richness as fixed effects and a nested random effect (~0+N| composition). In addition, we have changed the statistical analysis for the NBEF as well to allow a comparison of results. Please see the updated methods section (LL 638-650) and result section (LL 259-260 & 288-289), as well as the supplement Table S1.

Other comments:

Line 36: "extend" suggests that you used a partition that allows split stability contributions into complementarity and selection effects, which is not the case. Suggest to state: "Here we develop an approach to quantify NBES, inspired by / drawing on Loreau & Hectors's approach to assess ..."
We agree and have adjusted this accordingly. It now reads "Drawing from Loreau & Hector's[22] approach for quantifying the net biodiversity effect on ecosystem functioning, we here introduce an approach to assess the added stability provided by biodiversity in the face of disturbance and considering the multidimensional nature of stability." (LL45-48)

lines 55-61: I understand that these hypotheses apply to both simulations and experiments; however, at the start of the discussion you check whether they apply to either the experiment or the simulation, judging that there is some support when either is matched. Please evaluate your hypotheses for both simultaneously (only supported when both are met), matching paired hypotheses outlined in lines 55-61.
Thank you for raising this important point. We have updated the evaluation of the hypothesis and rejected H2 as a result, see Introduction LL 66-73 and Discussion LL301-312.

line 104: clarify/state how to standardise the response ratio
We added a sentence in LL 136-138 and hope it becomes clear now "The standardised response ratio (RR) thereby is calculated from the difference in community/ population biomass in a disturbed setting relative to an undisturbed (control) setting."

line 136: use trend or increasing temperature instead of "pressure"
Text revised as requested.

line 139: delete "significant" since this seems to be derived from visual rather than statistical inspection (also not recommending the latter!)
We thank the reviewer for noticing this and have removed the term.

line 140-141: "differed among disturbance types" seem to contradict the caption of Fig 2 where you state "only results from the increasing temperature are shown this disturbances regimes were similar"; did you mean variation in thermal optima?
We refer here to the results in the Supporting Information Figure S1. Because Fig. 2 in the main text is a part of this figure, we cited it here as well. To prevent confusion, we removed "Fig.2" here and refer solely to the supplementary figure, where different treatments for simulation runs with different temperature optima are compared.

lines 142-150 are rather descriptive and do not provide explanations for the observed patterns. Suggest to add some explanation for these patterns here or in the discussion.
We agree and have added a few sentences in the discussion LL326-337 to better explain observed patterns: "In the absence of thermal differentiation, stronger competition enhanced differences in species' biomass contributions, such that highly competitive species achieved higher biomass than expected, thereby elevating overall stability through increased mean performance[6,32,33]. In contrast, when species differed in their thermal optima, NBES became more variable and included neutral or negative effects, particularly under strong competitive interactions (Fig. 2). This variability may have arisen because differences in (thermal) traits have the potential to alter competitive hierarchies and reduce synchrony in population responses [34], leading to both stabilising and destabilising outcomes depending on how species' traits align with the prevailing temperature regime. In some cases, strong competition and high diversity in species temperature optima led to non-monotonic relationships between richness and biodiversity effects on stability (Fig.3 & Supporting Information Fig. S1)."

line 164: suggest to use only optimum/optima rather than "temperature preference(s)" for consistency here and throughout the ms
We agree and have now substituted preferences for optima here and throughout the MS.

line 306: do you mean Fig S5?
We have changed it to Fig S5, thanks for noticing.

line 382: Food webs are not an individual system (unless you refer to one specifically). Maybe refer to grasslands rather than food webs?
We were not referring to a specific system here but were hoping to inspire future research to look at net biodiversity effects on stability on a broader scale. We have rephrased the sentence to "Given the scale-dependency of biodiversity, it is essential to broaden the spatial and temporal scope of biodiversity research beyond individual systems (e.g., by using a food web design) and specific time frames." (LL 445)

line 445: did you discard observations to disregard the effect of transients in the simulations?
No, we did not discard any observations from the simulations when calculating NBES. This was because we wanted to be able to discuss experiment-based NBES dynamics in the context of simulated NBES dynamics where community traits were fully known, and vice versa. Since experiment-based NBES data take into account full system dynamics from the start of the experiment until the final sampling point, we calculated simulation-based NBES values in an analogous way to ensure full compatibility and comparability. Comparing simulation results in the treatment runs with control runs allows then to account for community-dependent transients.

Figures:

Fig 2:
Better to state alpha SD as the facet labels; only after reading the methods I realized that alpha refers to the SD and not the mean.
Fix "temperature" in panel title b)
Suggest to alert readers to the different Y axes scales: the positive NBES in panel a are consistent but much weaker than the NBES in panel b

Thank you for these important points. We changed the labels in Fig. 2 to "no competition", "intermediate competition" and "strong competition" to be accessible to a wider audience but provided details on alpha_sd in the figure caption. Additionally, we fixed the spelling error and added a note to the figure caption highlighting the difference in y-axis scale, as recommended.

Fig 3:
I have a hard time telling apart the 4 and 5 species communities in the experiments. Maybe use a different colour gradient or a different symbol for richness level 5?
In response, we chose a darker orange here for the 5 species communities and hope the distinction becomes clearer now. Also, we adjusted the calculation of the NBES by making the variation in the relative proportion of species in the control time dependent, instead of using a fixed value (i.e. relative proportion at the beginning of the experiment (t0), as in Hector and Loreau, 2004). The 5 species communities are displayed along the 0 line as there is only one 5-species mixture (Grand Mean of 5 spp community – Mean of community including spp A-T which is 1). Please see Box 1 for an updated calculation.

Fig 4:
Caption: Not clear what is meant by "indicate greater stability with positive and negative deviations?" Suggest only explaining what high and low observed OEV means in terms of stability.
In hindsight, the formulation is indeed not very clear, for which we apologise. We decided to remove the subsentence. The caption now reads: "Higher absolute values indicate greater instability that can result from positive and negative deviations in the treatment relative to the control."

Reviewer #2 (Remarks to the Author):

Key results and significance

This paper addresses the question of quantifying the effects of biodiversity on stability. This is a relevant and timely topic, and it is still understudied; therefore this study aims at filling an important gap. This goal is pursued by extending the framework developed by Loreau & Hector (ref. 19 in the manuscript) to asses the effect of diversity on ecosystem functioning. The authors define an index, the net biodiversity effect on functional stability (NBES), which quantifies the difference between observed and expected stability and can be computed starting from different metric of stability measurable by comparing a treatment response to a control. The NBES is then measured in simulations of a temperature-dependent Lotka-Volterra competition model and in microcosm experiment involving 5 diatom species grown in isolation and in all the possible combinations creating communities of 2, 4, and 5 species. The author reports that:

1. Simulated communities showed sometimes lower and sometimes greater stability than expected. Experimental communities showed overall greater stability than expected.

2. The NBES varied in direction and magnitude in the simulations. In the experiments, NBES was highest for two-species combinations and decreased at higher species richness levels.

3. The NBES varied among species combinations and temperature treatments according to species thermal optima in both simulations and the experiment, and even became negative for some.

4. In simulations, the magnitude of the NBES varied with competition strength.

The problem addressed by the authors is relevant and the approach is clear and potentially productive. I have, however, some major and minor comments that I detail below.

We are glad to hear that the reviewer found the manuscript to be relevant and thank the reviewer for their helpful and in-depth comments aimed at improving the mansucript. We have revised the calculation of the NBES approach and are now confident that it displays the added stability by diversity independent of the introduced interaction strength. Moreover, have added more text on the multidimensional nature of the OEV approach and added substantial text that hopefully better reflect the advantages of this approach while improving clarity.

Major comments

- The OVE is the main (in)stability proxy used in the paper, it has been devised to combine multiple dimensions of post-disturbance stability into a single measure of instability. Why this is the case, however, is not clearly described in the context of this work. While its operational definition is clear, its connections with different measure of instability is lacking. There are several references to reference 20, where it was introduced in 2021, but to the best of my understanding, it is not (yet) such a broadly used and known index that can afford to be carefully described in a work such as the present one. I think it is important to make a case also in the present work about how OVE explicitly connects to different dimensions of (in)stability and why it summarizes them meaningfully.

We agree that the relationship between OEV and other stability metrics should be better explained here and added a more detialed description to Box 1 "In this study, we assess the NBES primarily (though not exclusively) using the OEV index, as the OEV integrates the response over the whole system over time and therefore integrates a multitude of stability metrics[24]" (LL 127-130) and Methods "The OEV encompasses several dimensions of post-disturbance-stability[24] (cf. Supporting Information Fig. S7) and directly correlates with the effect size of the disturbance: A larger OEV indicates greater temporal variability, lower resistance to disturbance, slower resilience (recovery rate) and/or incomplete recovery[2,24]"(LL 596-599).

We also added the graph below to the supplement (Fig. S7), showing the correlation between OEV and temporal variability, measured as Coefficent of Variation of the response ratio over time (CV, a) and resistance, measured as the response ratio after initiation of the disturbance. Again, these results stress that OEV is indeed an integrative stability metric.

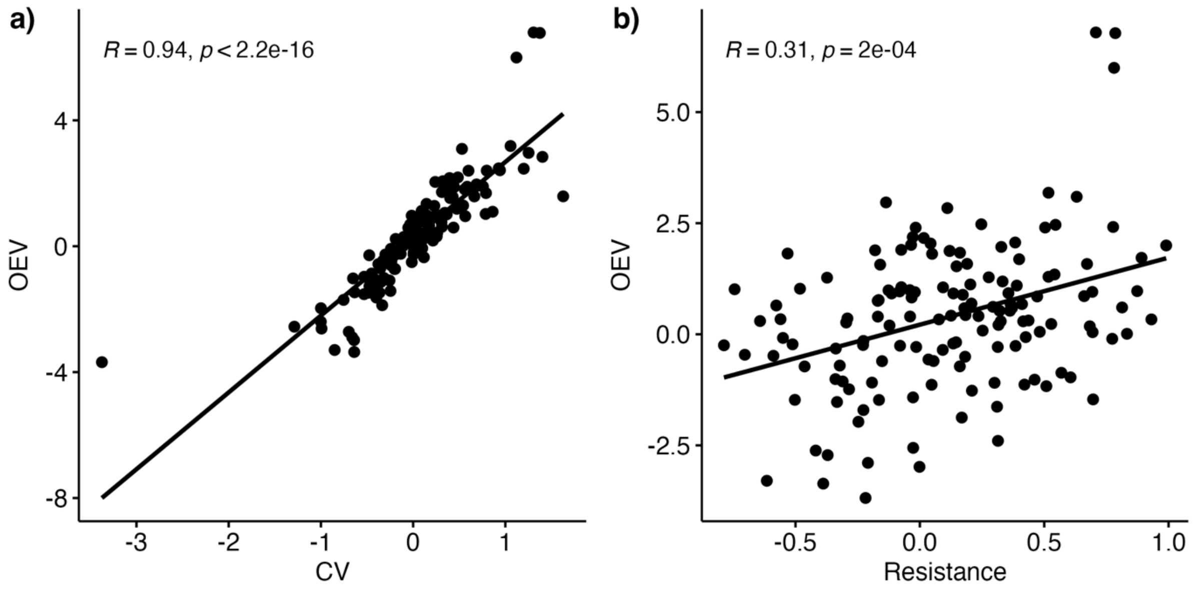

- The NBES results for the simulations in the case alpha=0 are potentially problematic, or, at best, hard to interpret. Following the model definition, the case alpha=0 correspond to an ensamble of non-interacting species. Therefore, by definition, biodiversity should not matter and should not show any net effect on stability. However, Figure 2 b and Figure 3 a and c show such effects, raising doubts on the reliability of the method and the interpretation of the NBES index.

We thank the reviewer for this excellent point and the careful assessment of this index. In response, we have adjusted the calculation of the NBES. Variation in NBES for communities without interactions resulted from not accounting for variation in the proportional abundances of species over time in the control. We now introduced such a time dependency which allows to clearly pin occuring patterns to the specific disturbance regimes and are confident that the results presented here now reflect underlying patterns. Please see also the updated description of $R_{con,i}$ in Box1 (LL106-111) that now reads “Relative biomass of species i in the control multi-species mixture (Y_(Con,i)) over time. Different to Loreau & Hector’s approach[22], we account for temporal dynamics in species relative proportions to separate the effects of disturbance treatments from naturally-occurring community dynamics.”

- I have some reservation on part of the interpretation of the data results on OVE and on NBES. Focusing on Figure 4 and on lines 193 to 197, while it is true that there is variability in the observed OVE, it does not seem the case that "the majority of multi species combinations showed positive or near-neutral deviations from the constant temperature controls". It seems to me that, while there is variability there are overall neutral deviations from the constant temperature control in the multispecies communities, with the exception of the case of 2 species where indeed there seems to be an overall positive trend. Regarding NBES (Figure 5), panel b for 4 species shows a dependence of the effects on the specific combinations rather than the overall number of species. For example the claim that the combined condition correspond to an overall posititve effect is biased by the high positive value of the combination ADGT, but it does not reflect the fact that actually for 3 combination out of 5 there is a negative effect.

We have rephrased this section in response to this comment, which did not fully reflect the results of figure 5 (please see LL235-242). The section now reads: “Observed stability showed considerable variation across treatments and richness levels as well as associated species combinations (Fig. 4). Most species monocultures showed negative deviations for

disturbances involving fluctuations, whereas gradual temperature increases led to positive deviations in some monocultures (Fig. 4). The majority of two-species combinations showed positive or near-neutral deviations from the constant temperature controls. In contrast, four-species combinations deviated both positively and negatively from their controls, with outcomes driven largely by which species were present rather than by richness itself.”

- Based on the results from the experiments, I do not fully agree with the conclusions regarding the 4 hypothesis that this paper addresses. H1 could be considered partly accepted, I agree, however, as pointed out in the previous comment the overall positive effect seen in Figure 5 a could be due to specific combination of species, rather than by the biodiversity itself. Regarding H2, "NBES increases with increasing species richness" I find a bit harder to consider it partly accepted, apart from the results that communities with two species seems to have a positive NBES, the observations seems to point towards the opposite, with this positive effect vanishing when the number of species increases. I am not fully convinced about H3. from Figure 3 b, d and e is not clear that the variation observed is due to thermal optima or other confunders. I am not necessarily suggesting that this is not the case, however there is space to improve on this point and clarify the arguments that lead to this conclusion in the result section. H4 is only addressed by simulation and therefore it should have a different (maybe lower) status with respect to the other hypothesis in my opinion.
Thank you for this important comment. We agree that the evaluation of the 4 hypotheses could be improved and have revisited them, as recommended. We now reject H2 and only partially accept H3, and we have added justification of these decisions in the text. We also added a paragraph on the effect of interspecific interactions on the simulation results to the discussion (LL326-337), which are of particular interest for hypotheses 3 and 4. It reads as: “In the absence of thermal differentiation, stronger competition enhanced differences in species’ biomass contributions, such that highly competitive species achieved higher biomass than expected, thereby elevating overall stability through increased mean performance[6,32,33]. In contrast, when species differed in their thermal optima, NBES became more variable and included neutral or negative effects, particularly under strong competitive interactions (Fig. 2). This variability may have arisen because differences in (thermal) traits have the potential to alter competitive hierarchies and reduce synchrony in population responses [34], leading to both stabilising and destabilising outcomes depending on how species’ traits align with the prevailing temperature regime. In some cases, strong competition and high diversity in species temperature optima led to non-monotonic relationships between richness and biodiversity effects on stability (Fig.3 & Supporting Information Fig. S1).”

Minor comments

- I believe the paper would benefit in clarity and impact if the experimental section is given more relevance by being discussed first in the results, with the simulations discussed only after it.
While we see the benefit of discussing the experimental results first to have a stronger focus on the experimental part, after exploring the merits of both approaches, we decided to keep the initial structure because experimental patterns, e.g. non-linearities for increasing temperatures but also the role of species traits, i.e. temperature optima, are explained using the simulations.

- It should be stated explicitly somewhere that the there are 1s on the diagonal of the interaction matrix of the Lotka-Volterra model.

We agree. We added the following “We assumed intraspecific competition in communities to be strongest. This is incorporated by setting the competition terms $\alpha_{i,j}$ along the diagonal of the competition matrix to 1, which is the maximum value of terms $\alpha_{i,j}$.” to LL 490-492 in the Methods.

Considerations on data and code availability, references to previous literature

Data and code are available and relevant previous literature is appropriately acknowledged. I do not have the expertise to judge the appropriateness and robustness of the experimental procedures.

Point-by-point responses

REVIEWERS' COMMENTS

Reviewer #1 (Remarks to the Author):

Overall, my concerns have been thoroughly addressed, and I feel that after this round of revision, the manuscript has improved both in clarity and in providing a more nuanced picture of the findings. This work provides an important advance for empiricists and theoreticians to understand the multifaceted relationships between species richness and traits mediating the stability of communities to climate change.

Response: We thank the reviewer for their valuable input that helped shaping the manuscript and are confident that we addressed all concerns.

When re-reading the manuscript, I noted a few minor (editorial) issues that should be fixed before moving forward.

Minor issues:
- Replace pressure with disturbance throughout the ms
Response: done

- lines 483-484: explain what you mean by "favourable optimum"? Small variation around a mean that is close to the expected average temperature change?
Response: We have rephrased the sentence: "When species shared a favourable optimum, that is all species had their temperature optimum at the mean temperature of temperature fluctuations (17.5 degree), NBES was consistently positive and increased with increasing competition strength. In contrast, greater variation in species thermal optima led to more variable NBES outcomes with much greater magnitude."

- Figure 2 panel b title: there is still a typo (Tempterature)
Response: We thank the reviewer for noticing and have corrected this.

- Figure 3: state more explicitly that the Y axis is the average bopt across communities, since the caption states that species had variable bopt
Response: Shown here is the species-specific influence on NBES calculated as the difference in NBES from the grand mean. Instead of species names on the y-axis we give the temperature optimum of the single species, as such species did have variable bopt.

- line 559: the figure suggests lower temporal stability; suggest deleting the statement in the parenthesis, which may be misinterpreted as higher temporal stability in the more species-rich communities.
Response: Done

- line 566: replace "gradual warming" with "temperature gradient of constant temperatures".
Response: Done

- line 589-593: This could also be interpreted oppositely: if you know the thermal optima of your community, you may be able to predict the community response given a know thermal regime and interaction structure. The composition effect is only idiosyncratic if you do not know the mapping between species identity and thermal optimum and the interaction structure, suggesting that predicting community responses to climate change crucially hinges on getting better estimates of these components across natural communities.

Response: We agree that composition effects are not inherently idiosyncratic in a mechanistic sense, as community responses should in principle be predictable given sufficient knowledge of species' thermal optima, interaction structure, and environmental regimes. However, in most ecological contexts these components are only partially known, such that even deterministic trait–environment relationships can generate outcomes that appear idiosyncratic at the level of observed community composition. Our results therefore highlight that predictability of community responses to climate change depends critically on the extent to which key mechanistic components of species' niches and interactions are resolved in natural systems.

Reviewer #2 (Remarks to the Author):

I appreciate the effort that the authors put into addressing my comments. And I am satisfied with all the reviews, except for one point.

I still believe that hypothesis 4 (H4) should notbe included with the others that have been explored experimentally, as it remains analyzed only through simulations. I expressed this concern, but it was not addressed.

I am not suggesting discarding the discussion on that point entirely. I think that the main hypothesis addressed formally should be the first 3, and a discussion about that aspect can find its place in the text while discussing the outcomes of the model, but not on the same level as the other hypotheses (in the intro and in the conclusions).

I think that rather than weakening the impact of the paper, clarifying this point will bring more clarity to what is being achieved and do a better service to the broader community.

Response: We thank the reviewer for their constructive feedback that has helped improving this manuscript. In response to the last comment and comments by the editorial office, we have now only predictions but not hypotheses. We agree that the distinction between the different predictions should be made clear and have rephrase this section as "For the simulations we further expect that the magnitude of the NBES depends on the strength of competitive interactions, given that interactions directly shape species responses to disturbances 27." By explicitly mentioning that we expect this only for the simulations, we are confidence that the importance of this expectation is lowered in comparison to the others.
